# Long-Term IGF1 Stimulation Leads to Cellular Senescence via Functional Interaction with the Thioredoxin-Interacting Protein, TXNIP

**DOI:** 10.3390/cells11203260

**Published:** 2022-10-17

**Authors:** Karthik Nagaraj, Rive Sarfstein, Zvi Laron, Haim Werner

**Affiliations:** 1 Department of Human Molecular Genetics and Biochemistry, Sackler School of Medicine, Tel Aviv University, Tel Aviv 69978, Israel; 2 Endocrinology and Diabetes Research Unit, Schneider Children’s Medical Center, Petah Tikva 49292, Israel

**Keywords:** insulin-like growth factor-1 (IGF1), IGF1 receptor, TXNIP, Laron syndrome, cellular senescence

## Abstract

The growth hormone (GH)–insulin-like growth factor-1 (IGF1) signaling pathway plays a major role in orchestrating cellular interactions, metabolism, growth and aging. Studies from worms to mice showed that downregulated activity of the GH/IGF1 pathway could be beneficial for the extension of lifespan. Laron syndrome (LS) is an inherited autosomal recessive disorder caused by molecular defects of the GH receptor (GHR) gene, leading to congenital IGF1 deficiency. Life-long exposure to minute endogenous IGF1 levels in LS is associated with low stature as well as other endocrine and metabolic deficits. Epidemiological surveys reported that patients with LS have a reduced risk of developing cancer. Studies conducted on LS-derived lymphoblastoid cells led to the identification of a novel link between IGF1 and thioredoxin-interacting protein (TXNIP), a multifunctional mitochondrial protein. TXNIP is highly expressed in LS patients and plays a critical role in cellular redox regulation by thioredoxin. Given that IGF1 affects the levels of TXNIP under various stress conditions, including high glucose and oxidative stress, we hypothesized that the IGF1–TXNIP axis plays an essential role in helping maintain a physiological balance in cellular homeostasis. In this study, we show that TXNIP is vital for the cell fate choice when cells are challenged by various stress signals. Furthermore, prolonged IGF1 treatment leads to the establishment of a premature senescence phenotype characterized by a unique senescence network signature. Combined IGF1/TXNIP-induced premature senescence can be associated with a typical secretory inflammatory phenotype that is mediated by STAT3/IL-1A signaling. Finally, these mechanistic insights might help with the understanding of basic aspects of IGF1-related pathologies in the clinical setting.

## 1. Introduction

The growth hormone (GH)–insulin-like growth factor-1 (IGF1) hormonal axis plays an important role in the regulation of metabolism, nutrition, cellular homeostasis, growth and aging [1,2,3]. Endocrine and tissue IGF1 concentrations are tightly controlled throughout development, typically reaching peak levels at puberty and being reduced during adulthood and aging [4]. Elevated dosages of IGF1 are associated with an increased risk of developing a number of malignancies, including breast, colon and prostate cancers [5,6,7,8,9]. Likewise, overexpression of the cell-surface IGF1 receptor (IGF1R) has been consistently linked to malignant transformation [10,11]. The enhanced expression of components of the IGF system in cancer reflects the inherent mitogenic, anti-apoptotic and pro-survival activities of this growth factor axis [12,13]. Consistent with the role of IGF1 in aging and longevity [14,15,16,17], late-life targeting of the IGF1R has been recently suggested to improve healthspan and lifespan [18].

Laron syndrome (LS) is a genetic disorder that results from the mutation or deletion of the growth hormone receptor (*GHR*) gene, thereby leading to impaired IGF1 production and dwarfism [19,20]. LS constitutes the best-characterized entity under the spectrum of congenital IGF1 deficiencies [21]. The typical features of LS are obesity, typical face, high basal serum GH and low to undetectable IGF1, which is unresponsive to the administration of GH [22]. Furthermore, epidemiological analyses have shown that LS patients are protected from cancer [23,24,25]. Recent genome-wide profiling of LS patients identified genes and signaling pathways that are differentially expressed in LS-derived lymphoblastoids in comparison to age-, gender- and ethnicity-matched controls [26,27]. Functional analyses using David and Web Gestalt bioinformatics tools allowed us to cluster differentially expressed genes according to their biological functions. Of interest, about 15% of the differentially represented genes were involved in metabolic pathways. Given the key role of IGF1 in the control of cancer cell metabolism, it is reasonable to assume that alterations in the expression and/or activity of IGF1-dependent metabolic genes may constitute an important mechanism in the establishment of a malignant phenotype [28].

Among other genes, genomic analyses identified thioredoxin-interacting protein (TXNIP) as one of the top upregulated genes in LS. TXNIP [29], also known as vitamin D3, upregulated protein-1 (VDUP-1) [30] or thioredoxin-binding protein-2 (TBP-2) [31], and plays a pivotal role in a number of cellular processes, including metabolism, growth, pyroptosis and apoptosis [32,33,34,35]. TXNIP regulates cell metabolism by inhibiting glucose uptake [36,37]. Additionally, TXNIP stabilizes the Cdk inhibitors, p16 and p27, which inhibit cell division and retain cells in the G_1_ phase [38]. These features help classify TXNIP as a member of the cell cycle inhibitors family. This role is supported by studies showing that TXNIP downregulation is a prerequisite for cell division [39,40] and that TXNIP functions as a *bona fide* tumor suppressor [41,42,43].

Given the involvement of the IGF1/insulin pathway in the regulation of metabolic processes, and in view of emerging information regarding the physiological and pathological activities of TXNIP [44,45,46,47], we recently examined the role of *TXNIP* as a downstream target for IGF1 action. Specifically, we reported the participation of TXNIP in IGF1R-mediated glucose metabolism and oxidative stress and identified a novel regulatory link between the IGF1 axis and TXNIP [48]. The ability of IGF1 to downregulate TXNIP under a number of stress conditions suggested that the anti-apoptotic effect of IGF1 is mediated via inhibition of TXNIP. In agreement with this notion, elevated TXNIP levels in LS may account for cancer protection in this pathology.

The present study was designed to evaluate the hypothesis that IGF1-stimulated cellular senescence is mediated via TXNIP activation. The rationale for this postulate lies in the fact that prolonged IGF1R activation can induce a senescence phenotype in normal cells through activation of the P53–P21 axis [49]. Accordingly, LS cells, which are exposed to lifetime low IGF1 dosages, might be less prone to become senescent or to efficiently handle a stress response—with ensuing cancer protection. In this study we show that: (i) IGF1-mediated TXNIP regulation is vital for maintaining cellular homeostasis; (ii) prolonged IGF1 treatment leads to the establishment of a premature senescence phenotype characterized by a unique senescence network signature; and (iii) combined IGF1/TXNIP-induced premature senescence is associated with a typical secretory inflammatory phenotype that is mediated by STAT3/IL-1A signaling.

## 2. Materials and Methods

### 2.1. Cell Lines

Human primary skin fibroblasts and mouse embryonic fibroblasts 3T3-L1 were maintained in DMEM supplemented with 10% fetal bovine serum (FBS), 2 mM glutamine and antibiotics. Human fibroblast cultures were generously provided by Dr. Carmit Levy (Tel Aviv University, Israel). The M12 prostate cancer-derived cell line was derived from the P69 benign prostate cell line by selection for tumor formation in nude mice. M12 cells are tumorigenic, highly metastatic and exhibit reduced IGF1 responsiveness [50]. The M12 cell line was a gift from Dr. Joy Ware (Virginia Commonwealth University, Richmond, VA, USA). In addition, human embryonic kidney cells (HEK293t) were employed. M12 and HEK293t cells were maintained in RPMI-1640 medium supplemented with 10% FBS, 2 mM glutamine and 50 µg/mL gentamicin sulfate. Reagents were purchased from Biological Industries Ltd. (Beit Haemek, Israel).

### 2.2. Generation of TXNIP Knock-Out Cell Lines Using CRISPR/Cas9

Guide RNA (gRNAs) sequences for CRISPR/Cas9 were designed on the CRISPR design website (on http://crispr.mit.edu/) (accessed on 20 February 2019) [51]. Insert oligonucleotides for human TXNIP gRNA #1 and #2 are 5′-CACCGGTAAGGAATATTCAACTCG-3′/5′-AAACTCGAGTTGAATATTCCTTACC-3′ and 5′-CACCGAATATGGGTGTGTAGACTAC-3′/5′-AAACGTAGTCTACACACCCATATTC-3′, respectively. The TXNIP guide RNA targets exon 3 of *TXNIP*. The complementary oligonucleotides for gRNAs were annealed and cloned into the pX458 CRISPR/Cas9-GFP vector (Cat. # 62988, Addgene, Cambridge, MA, USA). HEK293t cells were transfected with either pX458/gRNA #1 or pX458/gRNA #2 using the jetPEI reagent (Polyplus Transfection, Illkirch, France). HEK293t cells transfected with the PX458 vector (no guide RNA) served as a control. Twenty-four hours after transfection, GFP-sorted individual cells were grown into colonies, after which *TXNIP* sequences were analyzed by DNA sequencing and expression was analyzed by Western blots.

### 2.3. Overexpression Studies

A TXNIP expression vector was obtained from Addgene (# 18759). The GFP-TXNIP vector was constructed by fusing the N-terminus of mouse *txnip* in frame to a cDNA-encoding enhanced GFP, located in pEmd-C1 as the parental vector. HEK293t cells were transfected with either GFP-TXNIP or empty pEGFP-C1 using the jetPEI reagent. Twenty-four hours after transfection, media was replaced with fresh media. Overexpression of GFP-TXNIP was confirmed by Western blots. GFP fluorescence in monolayers was visualized using a fluorescence microscope under illumination at the 360–400 nm spectrum, which excites GFP fluorescence. Fluorescent images were obtained under 4× magnification on a Olympus microscope.

### 2.4. Real Time-Quantitative Polymerase Chain Reactions (RT-QPCR)

Two µg of total RNA was reverse transcribed using the Superscript^®^ First-Strand Synthesis System for RT-PCR (ThermoFisher Scientific, Waltham, MA, USA). Real-time–quantitative polymerase chain reactions (RT-QPCR) were performed using Faststart Universal SYBR Green Master (Rox, Roche Diagnostics GmBH, Mannheim, Germany). An ABI Prism 7000 Sequence Detection System was employed for these analyses. Amplifications were carried out after an incubation of 2 min at 50 °C and 10 min at 95 °C, followed by 40 cycles at 95 °C for 15 s, 1 min at 55 °C, and 30 s at 72 °C. Sequences of primers used to detect *TXNIP* mRNA were: TXNIP-F, 5′-ACTCGTGTCAAAGCCGTTAGG-3′; TXNIP-R, 5′-TCCCTGCATCCAAAGCACTT-3′. Sequences of primers used to detect *p53*, *p21*, *36B2* and *β-actin* were: P53-F: 5′- CACATGACGGAGGTTGTGA -3′; P53-R: 5′- CAAATTTCCTTCCACTCGGATAAG -3′; P21-F: 5′- GACACCACTGGAGGGTGACT -3′; P21-R: 5′- GGATTAGGGCTTCCTCTTGG -3′; 36B2-F: 5′-CGACCTGGAAGTCCAACTAC-3′; 36B2-R: 5′-ATCTGCTGCATCTGCTTG-3′; β-actin-F: 5′-CCTGGCACCCAGCACAAT-3′ and β-actin-R: 5′ GGGCCGGACTCGTCATACT-3′. Remaining primer sequences will be provided upon request. The number of PCR cycles performed to reach the fluorescence threshold was the cycle threshold (C_t_). Each cDNA sample was tested in triplicate and mean C_t_ values are reported. For each reaction, a ‘no template’ sample was included as a negative control. Fold-differences were calculated using the 2^ΔΔCt^ method [52].

### 2.5. Western Blot Analyses

Cells were grown to confluence, centrifuged for 10 min, washed twice with ice-cold phosphate-buffered saline (PBS) containing 5 mM EDTA, and incubated with a lysis buffer for 20 min. The suspension was centrifuged at 13,000× *g* rpm for 10 min and protein concentration was determined by the Bradford method (Bio-Rad Laboratories Ltd., Hercules, CA, USA). Aliquots (100 μg) were diluted with sample buffer, boiled for 10 min and resolved through 10% SDS-PAGE. Proteins were then blotted onto nitrocellulose membranes. After blocking, blots were incubated overnight with the indicated antibodies, washed, and incubated with a horseradish-peroxidase-conjugated goat anti-rabbit IgG (1:50,000; Jackson ImmunoResearch Laboratories, West Grove, PA, USA). Proteins were detected using the SuperSignal West PicoChemiluminescent Substrate (Pierce, Rockford, IL, USA). Tubulin expression was used as a loading control for total proteins.

### 2.6. Cell Treatments

A 50 mM stock solution of etoposide (Sigma-Aldrich, St. Louis, MO, USA) was prepared in DMSO. Cells were treated with 1, 5, 10 or 20 µM of etoposide for the indicated periods of time. Equal amounts of DMSO were added to control cells in each experiment. Ultraviolet (UV) irradiation was performed by removal of medium, washing once with PBS, and exposure to a controlled dose of UV (50 mJ/cm^2^) light using a UV cross-linker.

### 2.7. Cell Viability Assays

HEK293t wild-type and TXNIP-KD cells (5 × 10^3^ cells) were seeded in 96-well plates using serum-containing medium. After 24 h, cells were treated with 1, 5, 10 or 20 µM etoposide, or with the corresponding amount of DMSO (negative control). After 24 or 48 h, viability was determined by an XTT assay. In brief, a solution of XTT [2,3-Bis-(2-Methoxy-4-Nitro-5-Sulfophenyl)-2H-Tetrazolium-5-Carboxanilide] reagent was added along with the solubilizing solution and plates were incubated for 3 h. The colorimetric reaction was measured using an ELISA reader at a wavelength of 450 nm in three independent assays. Viability was expressed as a percentage of optical density values obtained in TXNIP-KD relative to empty vector-transfected cells. All experiments were performed in quadruplicate for three independent experiments.

### 2.8. Senescence-Associated β-Galactosidase Assays

Cells were washed twice with PBS and fixed with 2% formaldehyde/0.2% glutaraldehyde for 5 min. After two additional washes with PBS, 1 mL of staining solution per well [150 mM sodium chloride, 25.2 mM sodium phosphate, 7.36 mM citric acid, 5 mM potassium ferricyanide, 5 mM potassium ferrocyanide, 2 mM magnesium chloride and 1 mg/mL 5-bromo-4-chloro-3-indolyl-b-D-galactoside (X-gal), pH 6.0], was added to the cells and incubated at 37 °C for 2 h overnight depending on the intensity of the staining. The cells were again washed with PBS and photographed by bright field microscopy to count blue cells and phase contrast microscopy to count total cells. At least four fields (100× magnification, approximately 200–600 cells/field) were counted for each plate and at least two plates were counted for each condition (or cell type).

### 2.9. Prolonged IGF1-Mediated Senescence Induction

Cellular senescence assays were performed as described [53]. Briefly, cells were serum-starved for 96 h prior to treatment with IGF1 (50 ng/mL) or vehicle. Media (serum-free, low glucose DMEM) supplemented with IGF1 was replaced every 48 h. Six days after IGF1 treatment, cells were subjected to either protein or RNA isolation for Western blots or gene expression analyses, respectively.

### 2.10. Flow Cytometry Analysis for Apoptotic Cell Death

Cells were resuspended in 50–100 μL Annexin-V binding buffer (0.1 M Hepes pH 7.4, 1.4 M NaCl, 25 mM CaCl_2_) containing Annexin-V-FITC (#4700 MEBCYTO Apoptosis Kit) and propidium iodide (1 μg/mL). Cells were then analysed on a FacsCalibur system (Cytek Development Inc., Fremont, CA, USA).

### 2.11. Flow Cytometry-Based Cell Cycle Analysis

Cells were seeded in duplicate onto 6-well plates (10^4^ cells/well) for 24 h. Cells were then serum-starved for an additional 24 h and incubated in the presence or absence of IGF1 or insulin for 72 h. Cells were then washed with PBS, trypsinized and pelleted by centrifugation. The cells were permeabilized with Triton-X100, after which propidium iodide was added. Stained cells were analyzed using a FacsCalibur system.

### 2.12. LC-MS/MS-Based Proteomic Analyses

Cells were lysed in 6 M urea and 2 M thiourea in 100 mM Tris-HCl (pH 8.5). Ten ug of protein were reduced with 1 mM DTT for 30 min and alkylated with 5 mM iodoacetamide for 30 min in the dark. The lysates were diluted 4-fold with 50 mM ammonium bicarbonate, followed by overnight digestion with sequencing grade trypsin. Resulting peptides were acidified with trifluoroacetic acid. Peptides were resuspended in 2% acetonitrile/0.1% TFA prior to the LC-MS/MS analysis. Peptides were analyzed by liquid-chromatography using the EASY-nLC1000 UHPLC (Thermo Fisher Scientific) coupled with the Q-Exactive (QE)-Plus mass spectrometer. Peptides were separated on 75 μm i.d. × 50 cm long EASY-spray PepMap columns packed with 2 μm C18 beads with 100Å pore size. MS acquisition was performed in a data-dependent manner using the positive-ion mode—with a selection of the top ten peptides—and MS/MS analysis. Full MS spectra were acquired at a resolution of 70,000, *m*/*z* range of 300–1800 Th, with an AGC target of 3 × 10^6^ ions and maximal injection time of 20 milliseconds (ms). MS/MS spectra were acquired at a resolution of 17,500 with an AGC target of 1 × 10^5^. Dynamic exclusion was set to 30 s.

### 2.13. Data Analyses

Raw files of the MS were analyzed with the MaxQuant (Max Planck Institute of Biochemistry, Martinsried, Germany, version 1.5.6.9) integrated with the Andromeda search engine. MaxQuant parameters were performed using a label-free quantification algorithm (LFQ). Statistical analyses of the MaxQuant output tables were performed with the Perseus software. The dataset was filtered to retain only proteins with at least 70% of the samples with valid quantitative values. IGF1-treated primary human skin fibroblasts were categorized as ‘IGF1 treated’ group and compared to control serum-starved cells, i.e., ‘sfm’ group. Cells transfected with a TXNIP expression plasmid and treated with IGF1 were compared to IGF1-treated cells transfected with an empty plasmid. Student’s *t*-test was performed with an FDR threshold of 0.05. Enrichment analysis was performed on the *t*-test significant proteins using Fisher’s exact test (FDR 0.02) with KEGG, GOBP and GOMF annotations.

### 2.14. Statistical Analyses

Statistical package SPSS was used and graphs were produced using Prisma software. Non-parametric statistical methods (Mann–Whitney U test and Kruskal–Wallis test) were applied to determine the statistical significance of the differences between means. Parametric tests (Student’s *t-*test and ANOVA) were also used.

## 3. Results

### 3.1. TXNIP Maintains Cellular Homeostasis upon DNA Damage Stress

**Figure 1:**
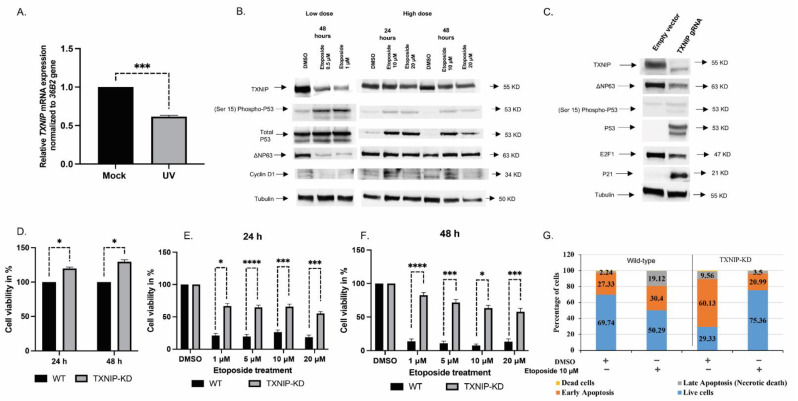
Analysis of the role of TXNIP in the DNA damage response. (**A**) Primary normal fibroblast cells (3 individual cell lines) were irradiated with UV (50 mjoule/cm), or left untreated, and harvested at 6 h for RT-QPCR analysis. *** *p* < 0.001 vs. respective untreated cells. (**B**) HEK293t cells were stimulated with low (0.5 μM and 1 μM) or high (10 μM and 20 μM) doses of etoposide for 24 or 48 h. After separation by SDS-PAGE, protein levels of TXNIP, P53, phospho-P53 (phosphorylated at serine 15), ΔNP63 and Cyclin D1 were determined using immunoblotting. Tubulin served as a loading control. Results shown are means ± S.D. of two independent experiments with duplicate samples. (**C**) HEK293t cells were maintained in full medium and transfected with a CRISPR/Cas9 plasmid containing a gRNA for the TXNIP gene (or non-targeting gRNA). Cells were sorted based on the expression of GFP. At the end of the incubation period, cells were harvested and lysed as described in Section 2. Lysates (100 µg) were analyzed by Western blotting for TXNIP, P53, phospho-P53 (phosphorylated at serine 15), P21, Cyclin D1, E2F1 and ΔNP63. Levels of tubulin were measured as a loading control. (**D**) TXNIP-KD HEK293t and wild-type cells were seeded in 96-well plates (3000 cells/well; four replicates) for 24 or 48 h, after which cell proliferation was assessed by XTT assays. A value of 100% was given to the cell number of wild-type cells. The experiment was repeated three times with similar results. (**E**,**F**) TXNIP-KD HEK293t and wild-type cells were treated with increasing doses of etoposide for 24 (**E**) or 48 (**F**) h, after which cell viability was measured by XTT assays. A value of 100% was given to the number of cells of DMSO-treated cultures. Shown is a typical experiment repeated three times with similar results. * *p*-value ≤ 0.05; *** *p*-value ≤ 0.001; **** *p*-value ≤ 0.0001 (**G**) Etoposide-treated wild-type and TXNIP-KD HEK293t cells were analyzed by flow cytometry to distinguish between apoptotic and necrotic cell death. Annexin V FITC/PI staining was used to assess the mode of cell death.

In previous studies, we identified the *TXNIP* gene as a downstream target for IGF1 action [48]. In order to study the effect of DNA damage on *TXNIP* expression, normal primary human skin fibroblasts were exposed to UV light at an intensity of 50 mJ/cm^2^ for 6 h, after which *TXNIP* gene expression was measured by RT-QPCR. The obtained results revealed that TXNIP mRNA levels were significantly downregulated upon UV treatment in three independent cell lines (Figure 1A). Next, we investigated the effect of the DNA damaging agent, etoposide, on *TXNIP* expression. At low (0.5–1 μM) doses, etoposide downregulated TXNIP levels. No effect, however, was seen at high (10–20 μM) doses, indicating that etoposide affects *TXNIP* gene expression in a dose-dependent fashion. P53 Ser15 phosphorylation and total P53 protein levels were markedly upregulated upon exposure to low doses of etoposide (Figure 1B). Additionally, we observed that low doses of etoposide reduced ΔNP63 and cyclin D1 expression. Taken together, these results suggest that TXNIP might play an important role in the DNA damage response.

To directly examine the effect of *TXNIP* expression on cell cycle regulatory proteins and stress response genes and, in a broad sense, to investigate the role of TXNIP in cellular homeostasis, we adopted the CRISPR/Cas9 technology. TXNIP silencing in pooled HEK293t-gRNA1 positive clones was confirmed by Western blots (Figure 1C). Next, we examined a series of classical stress-regulated genes, including P53, P63 and P21, in TXNIP-KD cells. The results of the Western blots indicate that total P53 expression as well as P53 Ser15 phosphorylation were markedly increased, leading, most probably, to activation of a cell cycle arrest transcriptional program via P21. In addition, we found that ΔNP63 expression was downregulated in TXNIP-KD cells. The reduction of ΔNP63 might explain the upregulation of P53 and P21 levels [54]. Finally, we observed downregulation of cell cycle regulatory protein E2F1 upon TXNIP silencing.

Consistent with a tumor suppressor role, we have previously shown that endogenous TXNIP expression is reduced in the M12 metastatic prostate cancer cell line relative to its benign counterpart, and that ectopic TXNIP overexpression in metastatic prostate cancer leads to cell death [48]. To assess the role of TXNIP in normal cell proliferation upon DNA damage, a colorimetric XTT assay was performed in TXNIP-KD HEK293t cells treated with increasing doses of etoposide (1, 5, 10, 20 μM) for 24 or 48 h. The results obtained indicate that TXNIP-KD cells proliferated more rapidly (~25%) than wild-type cells at both time periods (Figure 1D). Moreover, etoposide treatment reduced cell viability in a dose-dependent manner (up to ~80% reduction at a 20 μM dose after 48 h), and this suppressive effect was largely diminished in TXNIP-KD cells (~40% reduction). Hence, abrogation of TXNIP conferred enhanced resistance against DNA damaging agents upon cells (Figure 1E,F).

To obtain information on the role of TXNIP during the different stages of apoptosis under basal conditions as well as following etoposide treatment, we conducted a flow cytometry analysis of Annexin V/PI in wild-type and TXNIP-KD HEK293t cells. Under basal conditions, the portion of TXNIP-KD cells at early apoptosis was more than twice that of wild-type cells (Figure 1G). Treatment with 10 µM of etoposide for 48 h led to a small increase in the portion of cells at early apoptosis (from 27.3% to 30.4%) and a large increase in the portion of cells at late apoptosis (from 2.2% to 19.12%) in wild-type cells. On the other hand, etoposide treatment of TXNIP-KD cells led to major reductions in the portion of cells at both early (from 60% to 21%) and late (from 9.5% to 3.5%) apoptosis. In parallel, cells were lysed and proteins were extracted, quantified and immunoblotted against PARP antibodies. The results obtained show that PARP cleavage was more prominent in wild-type than in TXNIP-KD cells (Figure S1A,B). Thus, the intensity of the cleaved 89-kDa PARP fragment was significantly higher (46%) in wild-type cells compared to TXNIP-KD cells (Figure S1B). In addition, we overexpressed a low amount of a TXNIP-GFP-expressing fusion plasmid (1 µg) under the control of an eukaryotic promoter in wild-type and TXNIP-KD HEK293t cells. An empty-GFP plasmid was transfected in control cells. DMSO was used as a control for etoposide treatment. The results of this experiment show that the ectopic expression of TXNIP increased apoptosis in both wild-type and TXNIP-KD cells upon etoposide treatment (Figure S1C,D). The extent of this increase, however, was significantly larger in wild type cells. These results indicate that TXNIP-KD cells are resistant to etoposide treatment. In addition, PCNA levels were decreased and SP1 levels were increased upon etoposide treatment in TXNIP-KD cells compared to wild-type cells—possibly due to the increased phosphorylation of ATM (Figure S2). Given that PCNA levels reflect the rates of cellular proliferation by accumulating in the late G1 and early S phase, low levels of PCNA in TXNIP-KD cells further prove that these cells are predominantly accumulated at the S and G2 phases of the cell cycle.

### 3.2. The Mitogenic Effects of IGF1 and Insulin Are Amplified in TXNIP-Deficient Cells

**Figure 2:**
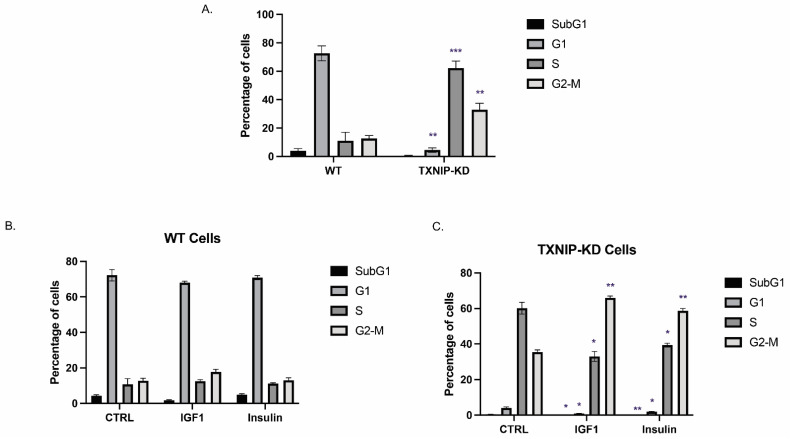
Effect of TXNIP on cell cycle distribution. (**A**) TXNIP-KD and wild-type HEK293t cells were starved for 24 h, after which they were subjected to cell cycle analysis using flow cytometry. Quantitative analysis of the proportion of the cells in each phase was performed from at least 10,000 cells per sample. (**B**,**C**) Wild-type (**B**) and TXNIP-KD HEK293t (**C**) cells were starved for 24 h, after which they were treated with IGF1 or insulin for an additional 24 h. Flow cytometry analysis of cell cycle shift by hormone treatment was then performed. * *p*-value ≤ 0.05; ** *p*-value ≤ 0.01; *** *p*-value ≤ 0.001.

Next, we explored the impact of TXNIP abrogation on cell cycle distribution. To this end, a flow cytometry analysis using propidium iodide staining was conducted on starved wild-type and TXNIP-KD HEK293t cells. As shown in Figure 2A, 61% and 33% of TXNIP-KD cells were accumulated in the S and G2 phases, respectively, compared to 11% and 13% in wild-type cells. The vast majority (71.35%) of the wild-type cell population accumulated in the G1 phase compared to only 4% in TXNIP-KD cells. Hence, the data are consistent with a scenario in which cells in the subG1 (4.2%) and G1 (71%) phases move towards the S and G2–M phase when TXNIP is absent, indicating that TXNIP is essential for maintaining G1/S cell cycle arrest. In agreement with the cell cycle data, the levels of E2F1 were markedly downregulated in TXNIP-KD cells (Figure 1C). Moreover, the decreased expression of Chk1 (Figure S2A) also indicates that TXNIP-KD cells probably accumulate in the S phase since Chk1 degradation happens during the replication (S) phase.

Next, we explored the effect of IGF1 or insulin on the cell cycle in cells with a silenced TXNIP. To this end, wild-type and TXNIP-KD HEK293t cells were treated with physiological (50 ng/mL) doses of IGF1 or insulin for 24 h following overnight starvation. IGF1 led to significant shifting of TXNIP-KD cells from the S phase (61% in control and 30% in IGF1-treated) towards the G2–M phase (33% in control and 66% in IGF1-treated) (Figure 2C). Insulin had a similar effect in these cells. Thus, cells from the S phase (61% in control and 38% in insulin-treated) shifted towards the G2–M phase (33% in control and 57% in insulin-treated). These results indicate that IGF1 and insulin significantly skewed a proportion of cells towards the G2–M phase in TXNIP-KD cells compared to wild-type cells. This phenomenon is particularly important in cancer cells, wherein TXNIP is often epigenetically silenced, thereby leading to a potentially amplified effect of both IGF1 and insulin on mitosis [29].

### 3.3. TXNIP Reactivation Upregulates E2F1 and Inhibits the IGF1R Signaling Pathway

**Figure 3:**
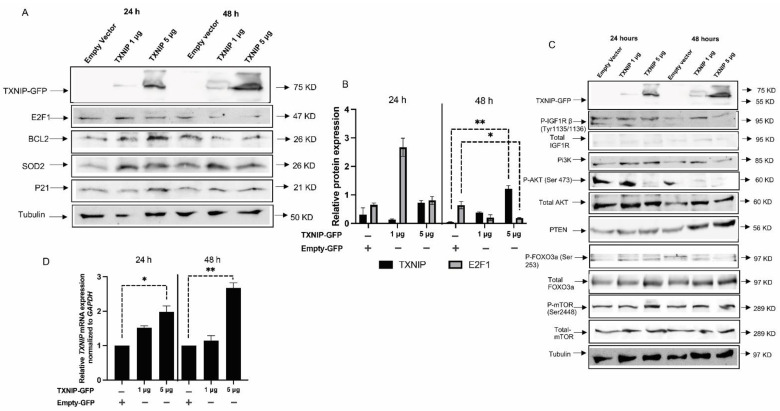
Effect of ectopic TXNIP overexpression in TXNIP-KD HEK293t cells. (**A**) TXNIP-KD HEK293t cells were transfected with 1 or 5 μg of a TXNIP-GFP expression vector (or empty plasmid) for 24 or 48 h. Cells were then harvested and levels of E2F1, BCL2, SOD2, P21 and tubulin were measured by Western blots. (**B**) Scanning densitometry analysis of TXNIP, SOD2 and E2F1 levels, as shown in panel A. (**C**) Western blot analysis of IGF1R/PI3K/AKT signaling mediators in TXNIP-GFP transfected cells. (**D**) RT-QPCR analysis of TXNIP mRNA levels in TXNIP-GFP-transfected cells. Values are normalized to the GAPDH housekeeping gene. Bars represent mean ± SEM of three independent experiments. A value of 1 was given to the mRNA levels in untreated cells. * *p*-value ≤ 0.05; ** *p*-value ≤ 0.01.

To understand the temporal and dose-dependent effect of TXNIP in normal cells, we re-expressed varying amounts of TXNIP-GFP in TXNIP-KD HEK293t cells for 24 and 48 h (Figure 3A,B); an empty PCDNA-GFP vector was used in control cells. Upon ectopic transfection of the TXNIP-GFP plasmid, the E2F1 protein was reduced at a dose of 5 μg of TXNIP plasmid, but there was no significant change in the levels of BCL2 and SOD2. These results are indicative of proper cell cycle regulation by TXNIP re-expression and, furthermore, suggest that the effect of TXNIP in cell cycle regulation is probably achieved via E2F1 downregulation.

As alluded to above, we identified TXNIP as a downstream target for IGF1 signaling; however, the specific role of TXNIP in this context is not clear. It is known, however, that TXNIP balances metabolic and growth signaling, thereby affecting PI3K signaling by PTEN disulfide reduction [55] and directly targeting AKT1. In order to understand the role of TXNIP in upstream and downstream pathways of AKT, we measured the levels of IGF1R targets in TXNIP-expressing and KD cells. Consistent with the literature, PTEN levels were increased along with AKT downregulation at 48 h upon transfection of 5 μg of TXNIP-GFP. Furthermore, FOXO3a levels were upregulated—possibly due to AKT downregulation—along with a reduction of FOXO3a Ser253 phosphorylation (Figure 3C). Interestingly, IGF1R activation was also reduced with no significant changes in mTOR levels. Figure 3D presents a quantitative graph of TXNIP mRNA levels in transfected cells. A quantitative analysis of IGF1R targets is presented in Figure S3.

### 3.4. TXNIP Augments the Effect of Prolonged IGF1-Induced Premature Senescence

**Figure 4:**
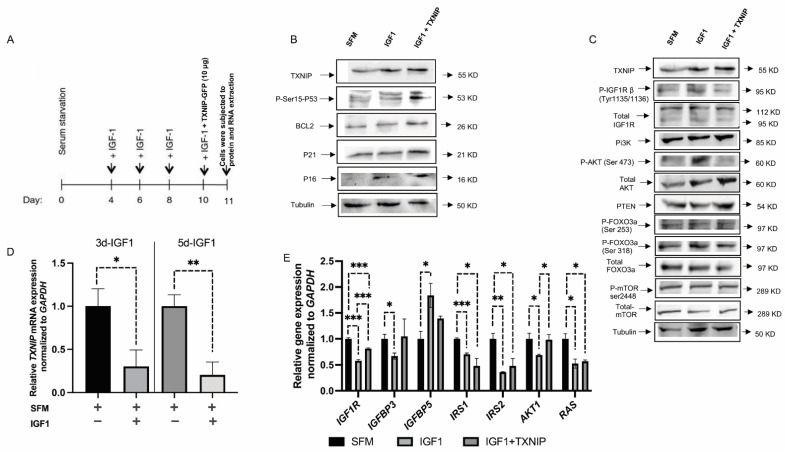
Effect of TXNIP on prolonged IGF1-induced senescence. (**A**) Schematic representation of senescence induction by prolonged IGF1 treatment. (**B**,**C**) Primary skin fibroblasts were treated with IGF1 for 11 days, transfected with a TXNIP-GFP vector during the last 24 h of IGF1 treatment, or serum-starved for the entire period and transfected with an empty pcDNA-GFP plasmid (SFM). At the end of the incubation period, cells were harvested and the indicated proteins were measured by Western blots. (**D**) Relative TXNIP mRNA levels upon 3 or 5 days of IGF1 treatment with IGF1 (or serum starved controls). (**E**) Gene expression analysis in IGF1-induced premature senescence with or without TXNIP-GFP transfection. GAPDH mRNA levels served as an internal control. * *p*-value ≤ 0.05; ** *p*-value ≤ 0.01; *** *p*-value ≤ 0.001.

**Figure 5:**
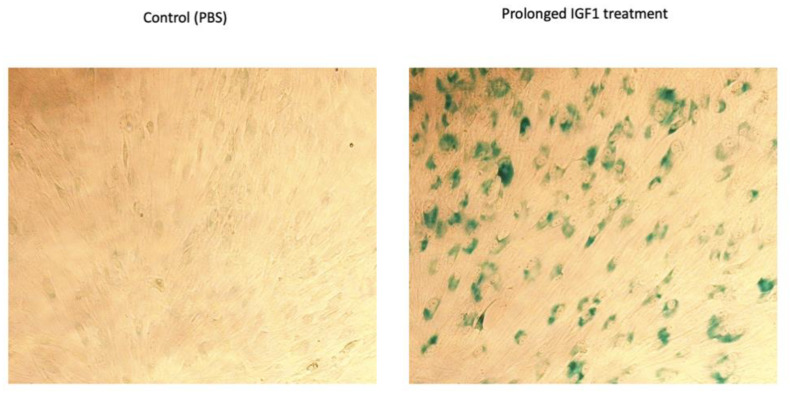
Analysis of β-gal staining upon prolonged IGF1-induced premature senescence. Representative images of β-gal staining in control (PBS-treated) and IGF-1 treated cells. Senescence was induced as described in Figure 4A.

It is generally accepted that IGF1 induces proliferation upon short-term incubation and cell cycle arrest upon prolonged exposure. To understand the role of TXNIP in IGF1-induced premature senescence, we utilized human primary skin fibroblasts. Low passage (P3) fibroblasts were grown in starvation medium and IGF1 was added after four days according to the protocol described in Figure 4A. After 7 days of IGF1 treatment (day 11 from the beginning), cells were lysed, and protein and RNA were extracted; control cells were starved for a total of 11 days. Our results show that TXNIP mRNA levels were upregulated 9-fold (Figure S4C) with a mild increase in protein levels (20%) upon long-term IGF1 treatment (Figure 4B). In agreement with our previous report, TXNIP mRNA was downregulated by 70% and 80% after six and eight days of IGF1 treatment, respectively (Figure 4D). In post-senescent cells treated for 10–13 days with IGF1, TXNIP levels were markedly reduced (Figure S4F). Increased levels of P21 and P16 reflect the induction of senescence by prolonged IGF1 treatment (Figure S4A). Beta-gal staining was performed, and the representative microscopic images are shown in Figure 5.

Given the role of TXNIP in maintaining cellular homeostasis in short-term regulation (Figure 1, Figure 2 and Figure 3), we asked whether TXNIP plays a role in prolonged IGF1-induced senescence. To this end, cells were transfected with 10 μg of TXNIP-GFP for 24 h after 6 days of IGF1 treatment (day 10 from the beginning); cells were then lysed and subjected to Western blot analysis. The results show that ectopic TXNIP expression increased P53 Ser-15 phosphorylation (220%), and increased the expression of BCL2 (300%), P21 (200%) and P16 (90%) (Figure 4B and Figure S4A). These results suggest that the effect of TXNIP on cell cycle arrest opposes the apoptotic role of TXNIP expression upon short-term induction.

Prolonged IGF1 treatment led to AKT activation (Figure 4C and Figure S4B) and the reduced expression of SIRT1, along with the reduced activation of ERK1 (Figure S4D,E). Interestingly, expression of the mitochondrial-maintenance-related gene, *SIRT3,* was upregulated upon TXNIP induction, whereas *SIRT1* levels were reduced (Figure S5A). In addition, we detected low levels of *IRS1*, *IRS2* and *RAS* mRNA expression upon prolonged IGF1 treatment compared to serum-starved cells (Figure 4E). In addition, upon ectopic TXNIP overexpression in IGF1-induced senescence, *P53* mRNA levels were increased compared to cells treated with only IGF1 (Figure S5A). To understand the link between IGF1, P53 and TXNIP, we utilized a P53-null endometrial cancer cell line (USPC2). The reduced TXNIP expression in USPC2 cells, in comparison to wild-type P53-expressing USPC1 cells, indicates that TXNIP is a downstream target of P53 (Figure S6). In addition, IGF1 treatment in both p53-null and p53-wild-type endometrial cells showed that the regulation of TXNIP by IGF1 is independent of P53 (Figure S8).

Interestingly, IGFBP3 was reduced upon prolonged IGF1-induced senescence, whereas senescence-associated IGFBP5 gene expression levels were significantly higher upon IGF1 treatment (Figure 4E). In order to test the potential alternative sources of energy consumption, we checked genes that are involved in glutamine metabolism. Interestingly, mitochondrial matrix enzyme glutaminase dehydrogenase (*Glud1*) levels were upregulated upon prolonged IGF1-induced premature senescence (Figure S5B). GLUD1 appears to function in both the synthesis and the catabolism of glutamate. TXNIP overexpression in IGF1-induced senescent cells led to the significant upregulation of *GLS2* mRNA levels—possibly due to P53-mediated transactivation, which can control glutamine metabolism (Figure S5B). Hence, ectopic TXNIP overexpression augmented the effect of prolonged IGF1-induced senescence in normal-skin fibroblasts. In contrast, the induction of senescence by etoposide or IGF1 in endometrial cancer cells is independent of P53 (Figures S7 and S8).

### 3.5. Proteomic Analysis Revealed a Unique Senescence Network Signature in IGF1-Induced Premature Senescence

To discover novel IGF1-induced senescence factors, including senescence-associated secretory phenotype (SASP)-related proteins, we established a streamlined proteomic workflow. To this end, proteins were collected from both IGF1-induced and IGF1/TXNIP-induced senescent primary human skin fibroblasts, along with quiescent cells. Senescence was induced by regular IGF1 treatment, as previously described, allowing ten days for the senescent phenotype to develop [49]. IGF1-treated cells were transfected with an empty-GFP plasmid as a control for TXNIP-GFP. The proteins were isolated and processed for MS analysis.

The use of a label-free data-dependent acquisition approach enabled a sensitive and accurate quantification of senescence-related SASP and other proteins. This unbiased proteomic profiling identified 2300 and 2487 proteins per sample in IGF1- and IGF1/TXNIP-treated cells, respectively. Proteins identified in senescent cells were quantitatively compared to controls, and significantly changing proteins with an FDR threshold of 0.05 were selected for further analysis. Each treatment and control group contained three biological replicates (see Section 2 for experimental design).

**Figure 6:**
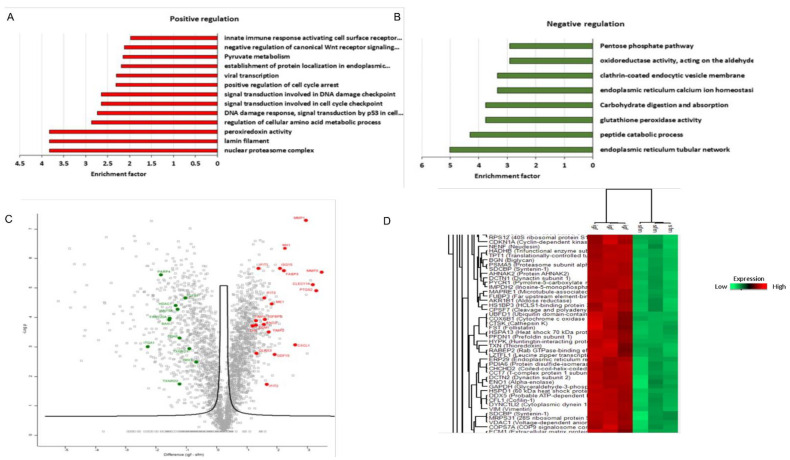
Signature of core senescence proteins, networks and pathways in prolonged IGF1-induced senescence. Summary of proteins significantly altered (*p*-value < 0.05) by prolonged IGF1 treatment compared to serum starvation condition in human skin primary fibroblasts. Analyses were performed with the Perseus tool using proteins with FDR-corrected *p*-value less than 0.05 and log_2_ fold-changes greater than 1.5. (**A**,**B**) Gene Ontology enrichment analysis of differentially expressed proteins in prolonged IGF1-treated cells. Proteins were ranked by average log_2_ fold-change and analyzed by gene set enrichment analysis (GSEA). Normalized enrichment score was calculated for upregulated and downregulated pathways resulting from prolonged IGF1 treatment. (**C**) Volcano plot representing average log_2_ (prolonged IGF1 treatment—serum starvation) fold-change in *X*-axis versus –log_10_ *p*-value in *Y*-axis, combined statistical significance from three datasets. Red and green dots denote proteins that were consistently up- or downregulated, respectively, in all three datasets (FDR-corrected *p*-value < 0.05, average absolute log_2_ fold-change > 1.5). (**D**) Hierarchical clustering representing a part of the cluster containing differentially expressed proteins with the smallest FDR-corrected *p*-value after integrating the three datasets. All independent technical replicates are shown. Note that the fold-changes presented in the figure are in logarithmic terms (log_2_). Log_2_ fold-change of 1.5 is equal to fold-change of 2 or (1.5)^2^.

A total of 573 and 864 proteins were shown to significantly change upon IGF1 or IGF1/TXNIP treatment, respectively [Fold-change cut off >2 (log_2_ value of 1)]. Notably, the top proteins after prolonged IGF1 induction were MMP3 [extracellular matrix (ECM) remodeling protein], PTGS2 (inflammation inducible COX-2) and CLEC11A (secreted growth factor) (9.3-, 8.3-, and 7.7-fold, respectively). This indicates that the cells were undergoing senescence by expressing inflammatory mediators [56] and growth factors, including ECM-related proteins (Figure 6A,C). Importantly, CDKN1A (P21) protein levels (3.8-fold) were increased in IGF1-induced premature senescence (Figure 6D). Interferon signaling, along with known SASP-related inflammatory markers—such as CXCL1, MX1, ISG15, MMP1, GDF15, etc.—were upregulated. The increased expression of a bona fide interferon-stimulating gene, MX1—along with ISG15—indicates an effective IGF1-induced, interferon-mediated senescence pattern that is similar to a protective anti-viral innate immune response [57,58] (Figure 6A,C). On the other hand, the downregulation of PARP4, TXNRD2 and HDAC1 indicates less efficient DNA repair and low levels of NAD^+^. Additionally, CDK6 (2.7-fold), a known keratinocyte senescence marker, and BAX (3.5-fold) were significantly downregulated in IGF1-induced senescence, which is indicative of cell cycle arrest (Figure 6B,C). Well-known SASP factors that were identified in the prolonged IGF1-treated fibroblasts include CXCLs, IGFBPs, matrix metalloproteinases (MMPs) and tissue inhibitors of metallopeptidase (TIMPs). Interestingly, IGF1R inhibition upon post-senescent cells led to the activation of P53 and Sting alpha signaling (Figure S9). A proteomic analysis of long-term IGF1-treated fibroblasts revealed a unique protein network along with previously identified SASP factors. Apart from the overlapping SASP-related protein expression, distinct interferon-mediated SASP factors were identified.

### 3.6. TXNIP Induction Drives a Distinct Senescence Phenotype in Prolonged IGF1-Induced Senescence

**Figure 7:**
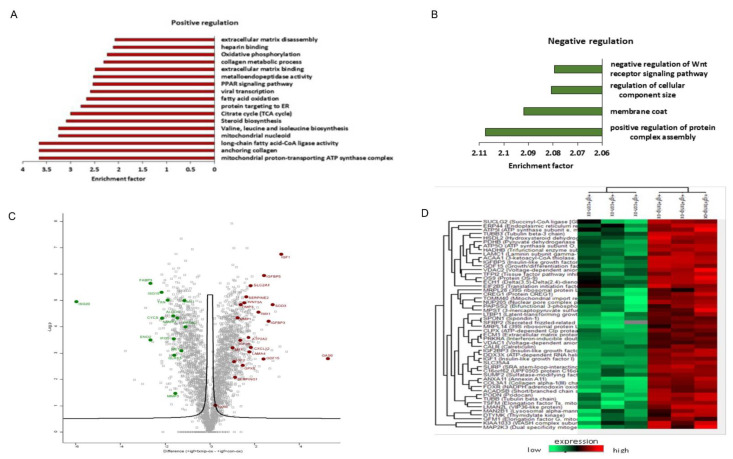
Signature of a unique senescence network and pathways upon TXNIP induction in prolonged IGF1-induced premature senescence. Summary of proteins significantly altered (*p*-value < 0.05) by TXNIP induction compared to empty vector-transfected in prolonged IGF1-treated human skin primary fibroblasts. Analyses were performed with the Perseus tool using proteins with FDR-corrected *p*-values less than 0.05 and log_2_ fold-changes greater than 1.5. (**A**,**B**) Gene Ontology enrichment analysis of upregulated (**A**) and downregulated (**B**) proteins in TXNIP-transfected prolonged IGF1-induced senescence. Proteins were ranked by average log_2_ fold-change and analyzed by GSEA, normalized enrichment score (NES) was calculated. (**C**) Volcano plot representing average log_2_ (TXNIP+IGF1—Empty vector+IGF1) fold-change in *X*-axis versus –log_10_ *p*-value in *Y*-axis, combined statistical significance from three datasets. Red and green dots denote proteins that were consistently up- or downregulated in all three datasets (FDR-corrected *p*-value < 0.05, average absolute log_2_ fold-change > 1.5). (**D**) Hierarchical clustering representing a part of cluster containing differentially expressed proteins with the smallest FDR-corrected *p*-value after integrating the three datasets. All independent technical replicates are shown. Note that the fold-change represented in the figure are in logarithmic terms (log_2_). Log_2_ fold-change of 1.5 is equal to fold-change of 2 or (1.5)^2^.

**Figure 8:**
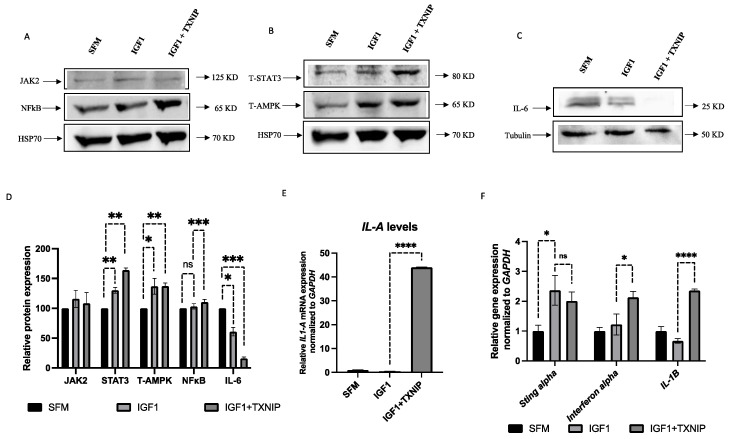
Effect of TXNIP on STAT3/IL-1A-mediated SASP in IGF1-induced senescence. (**A**–**C**) Primary skin fibroblasts were treated with IGF1 for 11 days (lane 2), transfected with a TXNIP-GFP vector during the last 24 h of IGF1 treatment (lane 3), or serum-starved for the entire period and transfected with an empty pcDNA-GFP plasmid (lane 1). Cells were then lysed and the levels of the indicated proteins were measured by Western blots. (**D**) Scanning densitometry analysis of JAK2, STAT3, NFkB, T-AMPK and IL-6 levels in IGF1-treated and IGF1-treated/TXNIP-transfected cells relative to serum-starved, empty vector-transfected cells. (**E**) RT-QPCR analysis of *IL-1A* mRNA levels in control, IGF1-treated and IGF1/TXNIP-treated fibroblasts. (**F**) RT-QPCR analysis of *STING-alpha*, *interferon-alpha* and *IL-1B* mRNA levels in control, IGF1-treated and IGF1/TXNIP-treated fibroblasts. * *p*-value ≤ 0.05; ** *p*-value ≤ 0.01; *** *p*-value ≤ 0.001; **** *p*-value ≤ 0.0001.

To determine how ectopic TXNIP expression affects the IGF1-induced senescence, we compared TXNIP to empty vector-transfected cells after prolonged IGF1 treatment. Strikingly, the senescence network was largely distinct from cells treated with only IGF1, with GAS6 (growth arrest specific gene 6), PXDN (peroxidasin homolog), LEMD2 (nuclear envelope protein) and IGF1 expression—with fold-changes of 38.5, 34, 10.7, and 9, respectively—as the top upregulated proteins (Figure 7A). Consistent with the gene expression results, IGFBP-5 and -3, in addition to IGFBP-2, were highly expressed. Highly represented proteins also included SOD3, GDF-15, Glut1 and Serpins (E2 and E1), which is indicative of IGF1 ligand stabilization and the energy-consuming senescence network. Given the Western blot results showing augmented IGF1R signaling inhibition by TXNIP induction (Figure 4C and Figure S4B), the proteomic results showed a significantly increased expression of IGF1 and IGF2 ligands—IGFBP-5, -3 and IGF2R (Figure 7C)—indicating a possible role for these growth factors as non-autonomous factors that influence neighboring cells. The upregulation of GPX8 in IGF1R downregulated conditions indicates that GPX8 might mediate an acute inflammatory response, which is consistent with its role in IGF1R-inhibited cells [59]. The increased expression of NDUFS2 and NDUFSB6 proteins hints at the activity of mitochondrial bioenergetics and ATP synthesis, which is consistent with the increased expression of AMPK (Figure 8B,D). Finally, redox-based ER quality control proteins TMX1, -2 and -4 were upregulated, which is indicative of a TXNIP-mediated ER stress response to the mitochondria (Figure 7A,C).

To determine whether there are core SASP pathways associated with the IGF1/TXNIP-induced premature senescence, we performed pathway and network analyses on overlapping proteins. The largest pathway associated with both treatments was related to common senescence-associated changes in tissue and cell structures, including ECM organization, laminin filament proteins, high mobility nuclear proteins, coagulation factors, inflammatory response proteins and metalloproteinases. Strikingly, interferon-stimulated genes that were upregulated upon IGF1 treatment were downregulated by TXNIP. These genes include ISG20, MX1, ISG15 and IFT35 (Figure 7B,C). The downregulation of nicotinamide phospho-ribosyltransferase (NAMPT) levels (−2.7 fold-change) by TXNIP might be indicative of augmented NAD^+^ reduction in prolonged IGF1-mediated senescence (Figure 7B,C). Of note, cytosolic thioredoxin (TXN1) levels were significantly downregulated, whereas mitochondrial TXN2 was unchanged. These results indicate that endogenous TXNIP possibly interacted with cytosolic TXN1, which was already upregulated upon prolonged IGF1-induced senescence. The downregulation of specific metabolic genes (FABP3, ENO2, ME1 and ALDOC) by TXNIP transfection indicates a distinctive metabolic phenotype from IGF1-induced senescence. Finally, WNT5a, a potent inflammatory factor that can act in autocrine/paracrine manners, was upregulated upon TXNIP induction (+2.8-fold-change), whereas the protein levels of WNT5a were low (−2.8-fold-change) in IGF1-induced senescence. Overall, these proteomic analyses identified unique Senescent Cell Apoptotic Pathways (SCAPs) and SASP signatures in prolonged IGF-induced senescence, which was augmented by TXNIP induction with a distinct SASP and metabolic signature.

### 3.7. TXNIP Induces a STAT3/IL-1A-Mediated Senescence-Associated Secretory Phenotype (SASP) in IGF1-Induced Senescence

Senescent cells secrete a distinct set of factors, collectively termed SASP, which have been postulated to carry both pro- and anti-tumorigenic properties, depending on the tissue context. An analysis of SASP-related genes in IGF1/TXNIP-induced premature senescent cells identified a secretory inflammatory phenotype mediated by STAT3/IL-1A signaling. Firstly, prolonged IGF1 exposure leads to the activation of energy sensor AMPK along with JAK2/STAT3 inflammatory-phenotype-related proteins, which were mildly activated (~30%) (Figure 8A,B,D). Interestingly, TXNIP overexpression leads to the upregulation of STAT3 by 50%, along with significant IL-6 reduction (Figure 8C,D). Finally, a gene expression analysis of other inflammatory cytokines revealed that the TXNIP induction in IGF1-induced senescence leads to a 40-fold increased expression of *IL-1A* (Figure 8E), which controls the late arm of the senescence secretome [60]. In addition, TXNIP augments *interferon α* and *β* mRNA levels but causes no significant changes in *STING (stimulator of interferon genes)-alpha* levels (Figure 8F), which has been identified as a key player that is responsible for coupling the sensing of DNA to the induction of powerful innate immune defense programs. Interestingly, abrogation of IGF1R by selective inhibitor AEW-541 (Figure S9A) during prolonged IGF1 induction leads to the upregulation of *STING-alpha* (Figure S9B), which indicates that the IGF1 pathway, but not TXNIP, is involved in the upregulation of STING-alpha signaling during senescence. These results suggest that the inhibition of IGF1R/AKT-induced premature senescence by TXNIP augments P53-mediated cell cycle arrest. In addition, distinct IL-1A-mediated SASP network activation depends solely on IGF1 signaling—not TXNIP signaling.

## 4. Discussion

### 4.1. Identification of Functional Interactions between TXNIP and the IGF1 Pathway

The involvement of IGF1 signaling in aging and cancer biology has been the focus of extensive research for several decades. Elevated endocrine IGF1 has been consistently associated with an enhanced cancer risk, whereas IGF1 levels are reduced during aging [17,28]. Multiple studies have shown the beneficial role of IGF1 downregulation in various species from worms and Drosophila to mice [15,61,62]. Our previous study on Laron syndrome, a rare congenital IGF1 deficiency, identified a novel link between the IGF1 signaling pathway and TXNIP [48]. In the present study, we dissected the role of IGF1-mediated TXNIP expression and action under various stress-induced cell fate decisions.

Our results show that, along with P53 and P21 upregulation, TXNIP was downregulated upon UV-induced DNA damage. This data corroborated the results from a recent study showing that UV-light exposure led to extensive changes in the expression of metabolic genes, including the significant downregulation of TXNIP [63]. In most types of cells, low doses of etoposide usually induce senescence, whereas higher doses lead to apoptosis [64]. In our study, we showed that low doses of etoposide downregulated TXNIP protein expression along with P53 and P21 upregulation. On the other hand, CRISPR/Cas9-mediated TXNIP knockdown leads to increased levels of P53 and P21. Given that the overexpression of TXNIP activates the apoptosis cascade pathway [47,65] we hypothesized that loss of TXNIP might disturb the homeostatic balance and lead to abrogation of apoptosis, cell cycle arrest or senescence under conditions of growth stimulation or various stress stimuli [66]. Indeed, our results show that TXNIP-KD cells proliferate faster than control cells even under etoposide treatment. Of interest, a dominant-negative form of P63 (ΔNp63), and E2F1 levels, were markedly downregulated in TXNIP-KD cells.

We have previously reported that the basal expression of *TXNIP* was markedly reduced in metastatic prostate and breast cancer cell lines compared to their benign counterparts, and that the overexpression of TXNIP inhibited the migration of androgen-independent PC3 prostate cancer cells [48]. Consistent with these findings, we show now that TXNIP-KD cells proliferate at a faster rate, along with reduced apoptosis—as indicated by the reduced cleavage of PARP1. These results show that TXNIP might play an important role in maintaining cellular homeostasis after DNA damage caused by stress. In the context of cell cycle regulation, we provide evidence that, under stress conditions, TXNIP mediates cell cycle arrest, mainly in the G1 phase. These results corroborate previous reports [38,39,67]. On the other hand, IGF1 signaling pathways are evolutionarily conserved to promote cell cycle progression in multiple species [68]. Hence, we showed that IGF1 and insulin significantly augmented the proportion of cells at the G2–M phase in TXNIP-KD cells. These results are particularly relevant in cancer settings where the pro-tumorigenic effect of IGF1 in TXNIP-silenced cancer cells might further lead to rapid cell division [69]. Of notice, even insulin can elicit a mitogenic effect in the absence of TXNIP.

The results from the TXNIP reactivation experiments revealed the role of TXNIP in cell cycle control via the downregulation of E2F1 levels. Studies showed that deregulated E2F1 induced hyperplasia and senescence-like features [70]. Ectopic TXNIP expression in TXNIP-KD cells also resulted in IGF1R downregulation, AKT inhibition and FOXO3a stabilization, along with PTEN upregulation. These changes in FOXO3a and PTEN levels suggest that TXNIP might mediate the regulation of ROS activity [71,72]. Taken together, the data indicate that TXNIP helps in regulating the process of cell cycle arrest and apoptosis upon cell fate decision. Furthermore, these findings reflect the role of TXNIP in attenuating the IGF1R signaling pathway in different ways. These complex interactions led us to investigate the role of TXNIP in prolonged IGF1-induced premature senescence.

### 4.2. Prolonged IGF1-Induced Cellular Senescence Upregulates TXNIP Levels

Cellular senescence is regarded as a cell state that is implicated in a number of physiological processes as well as a spectrum of age-related diseases. Senescence is accompanied by senescence-associated growth arrest (SAGA), which usually includes a senescence-associated secretory phenotype (SASP). The targeting and elimination of senescent cells in cancer therapy and age-related disorders have been suggested to be a potentially efficient method for improving lifespan in parallel with targeting IGF1R signaling [73,74,75,76,77]. Of interest, a recent study identified GH as a component of SASP that is often activated in senescent cells, promoting an aging phenotype via paracrine and autocrine mechanisms [78]. IGF signaling is known to induce senescence upon chronic IGF1R activation [49] but the unique secretory features of senescence by IGF1 induction are largely unknown. IGF1 signaling plays a dual role by inducing mitogenic and survival effects upon short-term exposure and premature senescence upon long-term treatment. Our results further show that prolonged IGF1-induced cellular senescence upregulates TXNIP levels.

Interestingly, the ectopic expression of TXNIP augments the effect of IGF1-induced senescence by upregulating the levels of BCL2 and downregulating the IGF1R pathway, which is indicative of distinctive Senescent Cell Anti-Apoptotic Pathways (SCAPs). Our data also show that: (1) *SIRT1* and *P53* mRNA levels are downregulated in IGF1-induced premature senescence; and (2) TXNIP overexpression leads to the reactivation of *P53* mRNA, thereby returning it to normal levels. Our data also show that IGF1-induced premature senescence reduces *IGF1R*, *IRS1* and *IRS2* mRNA levels, thus reflecting the downregulation of IGF1R signaling. The results from glutamine-synthesis-pathway-related gene expression shows that the upregulation of *GLUD1* mRNA is involved in IGF1R-mediated premature senescence. Targeting glutamine synthesis is currently an important strategy in the area of senolytics and cancer therapy [79,80]. Ectopic TXNIP overexpression in prolonged IGF1-mediated senescence leads to the increased expression of *GLS2* mRNA levels, which reflects the stabilization of P53 by TXNIP as GLS2 is a direct target of P53 activation [81]. Finally, the augmentation of IGF1-induced premature senescence by TXNIP indicates that TXNIP downregulates IGF1/AKT signaling and stabilizes P53 in order to induce effective cell cycle arrest by acting as a transformation barrier [82].

### 4.3. Proteomic Analyses Reveal Activation of a Pro-Inflammatory Network in IGF1/TXNIP-Induced Premature Senescence

In senescent cells, the survival network also includes a secretory phenotype, which is controlled by various factors, mainly by the JAK/STAT and NF-kB pathways. These paths can initiate the activation of a number of interleukins and CXCR2-binding chemokines [83]. SASP regulates neighboring cells by secreting non-autonomous factors, including both growth and inflammatory molecules. These factors are heterogeneous, given that their differences are dependent on the cellular context and the mode of senescence induction [84]. These paracrine factors can reproduce the SASP and amplify the secondary activation of these factors [85]. In our study, prolonged IGF-induced senescence activates JAK2/STAT3 levels but, intriguingly, IL-6 levels are reduced. Moreover, TXNIP induction leads to increased activation of STAT3, which is indicative of a unique STAT3-mediated signaling pathway that is independent of IL-6 activation. Interestingly, TXNIP induction leads to the increased expression of *IL-1A* gene expression, which is consistent with the activation of a potent pro-inflammatory SASP arm [60].

Other important features of senescent cells are mitochondrial dysfunction and deregulated energy metabolism, which are associated with reduced NAD^+^ levels. AMPK is activated during most of the senescence process, including during mitochondrial dysfunction in which ATP levels are low. As a corollary, the NAD^+^/NADH ratio is reduced in the senescent cells [86]. Our results show that prolonged IGF1-induced premature senescence leads to the increased expression of AMPK. Recent studies identified a unique senescence network mediated by mitochondrial dysfunction that is mainly controlled by the AMPK/P53 network with reduced SIRT3 and NAD^+^ levels [87]. In our study, the IGF1-induced premature senescence network displays reduced SIRT1 along with increased AMPK and P53, indicating that these senescent cells might have dysfunctional mitochondria. On the other hand, TXNIP induction leads to the activation of *SIRT3* levels, indicating a partial recovery from mitochondrial dysfunction with a unique metabolic phenotype.

A recent study has shown that chronic senescent cells can also activate the L1 transposon, which can initiate an innate immunity-mediated interferon response as a protective response to endogenous viral element activation [88]. This inflammatory response activates a number of interferon-related genes [89]. Our proteomic analysis results are intriguing as the top proteins expressed in prolonged IGF1-induced premature senescence express various interferon- and inflammatory-related proteins. Specifically, our data show that prolonged IGF1 treatment seems to balance STING-alpha activation while upregulating interferon signaling. Some of the highly expressed proteins are predominantly interferon-inducible factors, including MX1, ISG15, ISG20, HERC5, IFIT1, IFIT2, IFIT3, OAS1, GBP1, IRF2BP2, etc. The senescence-associated proteins found to be highly expressed upon prolonged IGF1-induced senescence include CDKN1A (P21), IGFBP-5, GDF-15, MMP-3, ECM, TIMP1, TIMP2, CXCL1, SERBP1, etc. These proteins are commonly expressed upon induction of senescence by various specific stimuli, including oncogene-induced senescence, replicative senescence and DNA damage-induced senescence [90,91]. Some of these proteins were also reported to be involved in pro- and anti-tumorigenic transformation by paracrine action [92]. Consistently, our gene expression analysis showed a similar upregulation of *IGFBP-5* levels. On the other hand, proteins related to the cell cycle and apoptosis are downregulated, like BAX and CDK6. Of note, CDK4/6 inhibition leads to a P53-mediated secretory phenotype (PASP). Furthermore, NAD^+^-consuming enzyme, PARP4, levels are reduced, indicating a low NAD^+^ level [93]. Finally, PPAR-γ co-activator, FAM120A, is downregulated along with the downregulation of TGF-β, Wnt5A and integrin-related proteins, indicating a distinctive senescence phenotype in prolonged IGF1-induced senescence.

### 4.4. Proteins Involved in IGF1 Signaling Are Upregulated upon TXNIP Induction

The mechanisms responsible for oncogenic transformation are still poorly defined [94,95]. Various forms of tumor induction in normal cells predominantly lead to senescence, mainly ras/myc-mediated senescence [96]. On the other hand, pharmacological inhibition of mTOR attenuates replicative cellular senescence [97] and also extends healthy aging to some extent [98]. The fact that ras and myc inhibit TXNIP expression [99,100] while rapamycin upregulates it [101] led us to hypothesize that TXNIP might play an important role during the initiation of cancer or induction of senescence by metabolic reprogramming. Our gene expression analysis showed a distinct SASP-related expression of IL-1A upon TXNIP-induced IGF1-mediated premature senescence. In addition, proteomic analyses revealed TXNIP-induced downregulation of most of the interferon-inducible genes that were upregulated in IGF1-induced senescence, including MX1, ISG20, ISG15, OAS1, IFIT3, IFIH1, etc. As expected, the highly expressed proteins include GAS6, Serpin G1, Serpin E2, CXCL12, TIMP3, LAMA4, etc., which are known senescence markers. Of note, Gas6 plays a critical role in inhibiting the innate inflammatory cytokines by inhibiting IL-6 [102] and activating the IL-1A-mediated inflammatory response [103], which is consistent with the reduced expression of IL-6 and overexpression of IL-1A. The common proteins shared with IGF1-induced senescence include IGFBP-5, GDF-15, etc., which is consistent with our data on their involvement in the senescence-related pathway [104,105]. Finally, many proteins involved in IGF1 signaling are upregulated, including IGFBP-3, IGF2 and IGF2R, indicating a stabilization of the IGF1 ligand, which is possibly due to the inhibition of the IGF1R signaling pathway by TXNIP induction [106].

The fundamental mechanisms governing cell fate decisions between growth and cell cycle arrest/cell death depend on multiple biochemical, molecular and genetic conditions and factors. In this respect, it is important to understand the effect of long-term IGF1 induction. Moreover, it is also intriguing to test the role of TXNIP in the prolonged IGF1-induced senescence phenotype since TXNIP is highly expressed in the late stages of aging and diabetes as an inflammatory marker and, conversely, as a tumor suppressor in cancer. A schematic comparison of short- versus long-term effects of IGF1 is shown in Figure S10. Overall, our results on prolonged IGF1/TXNIP-mediated senescence show that prolonged IGF1-mediated premature senescence features a pro-tumorigenic and innate immunity activation program, which can be switched to a unique IL-1A-dependent augmented senescence phenotype by TXNIP induction. This inflammatory response, however, is not directly due to TXNIP but rather due to the inhibition of IGF1R signaling. Taken together, these findings shed light on fundamental aspects of tumor initiation and inflammation in aging, cancer, diabetes and other age-related disorders. In addition, these mechanistic insights might help with the diagnosis and treatment of age-related illnesses in the clinical setting.

## Data Availability

Not applicable.

## Supplementary Materials

The following supporting information can be downloaded at: https://www.mdpi.com/article/10.3390/cells11203260/s1, Figure S1: Effect of etoposide treatment on apoptosis-related PARP cleavage; Figure S2: Effect of etoposide on DNA damage related protein expression; Figure S3: Scanning densitometry analysis of the effect of TXNIP expression on the IGF1 signaling pathway; Figure S4: Effect of prolonged IGF1 treatment, or IGF1+TXNIP treatment, compared to untreated serum starved cells, on signaling mediators; Figure S5: Effect of prolonged IGF1, or IGF1+TXNIP, treatment on selected gene expression; Figure S6: Effect of TXNIP KD in p53 mutated uterine cancer cells; Figure S7: Effect of etoposide treatment on senescence induction in USPC1 and USPC2 uterine carcinoma cells; Figure S8: Effect of IGF1 on TXNIP expression in USPC1 and USPC2 cells; Figure S9: IGF1R activation sustains the inhibition of STING-alpha activation during prolonged IGF1-induced premature senescence; Figure S10: Schematic diagram of transient versus prolonged IGF1 treatment.
