# Supplementary material for: Long-Term IGF1 Stimulation Leads to Cellular Senescence via Functional Interaction with the Thioredoxin-Interacting Protein, TXNIP
Source: Cells. 2022 Oct 17;11(20):3260. doi: 10.3390/cells11203260 (PMC9601129; doi:10.3390/cells11203260)
Supplement: Supplementary file 1 [file cells-11-03260-s001.zip › cells-1913062-supplementary.pdf]

Figure S1

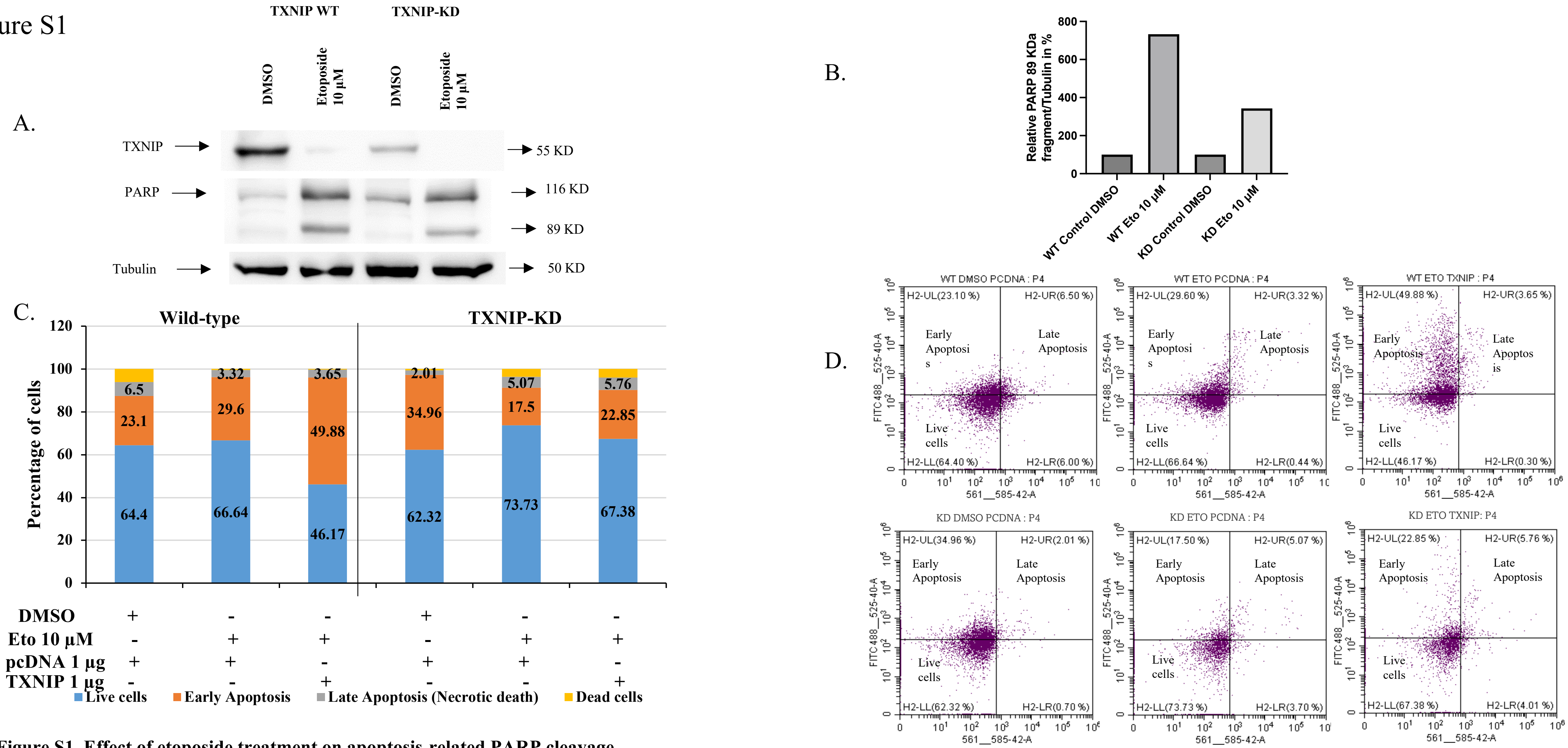

Figure S1. Effect of etoposide treatment on apoptosis-related PARP cleavage.

(A) Control and TXNIP-KD HEK293t cells were treated with etoposide (or DMSO, control) for 48 h, after which PARP cleavage was assessed.

(B) Scanning densitometry analysis of the 89-kDa (cleaved) PARP fragment. Bars denote intensity of PARP bands normalized to tubulin.

(C) Control and TXNIP-KD HEK293t cells were transfected with a TXNIP-GFP expression plasmid (or empty vector), after which cells were treated with etoposide (or DMSO). Verification of apoptotic or necrotic cell death was done by flow cytometry using Annexin V FITC/PI staining.

(D) Analysis of Annexin V FITC/PI staining. Each panel represents the sorting of cells depending on Annexin V and propidium iodide staining. For each graph, left bottom quadrant represents live cells, top left and right quadrants represent early and late apoptosis, respectively, and bottom right quadrant represents dead cells (in %). The three upper graphs represent wild-type cells (three left columns in panel C) and the three bottom graphs represent TXNIP-KD cells (three right columns in panel C).

Figure S2

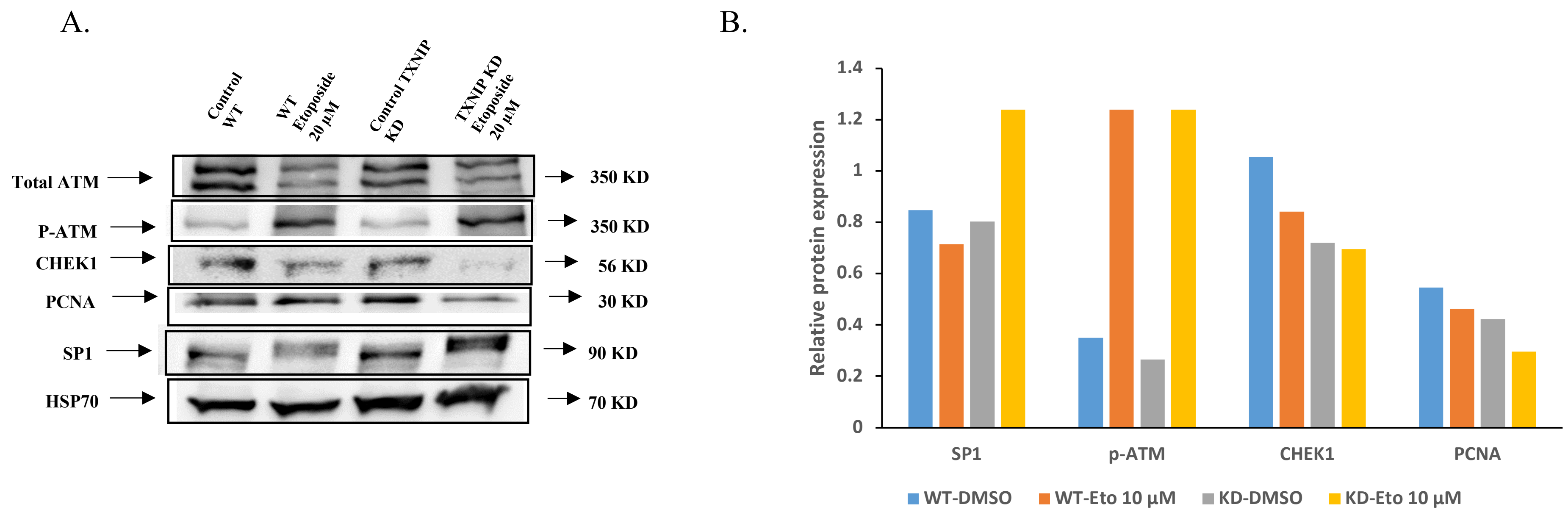

Figure S2. Effect of etoposide on DNA damage related protein expression.
(A) Control and TXNIP-KD HEK293t cells were treated with etoposide (20 µM) (or DMSO) for 24 h, after which cells were harvested and levels of total- and phospho-ATM, Chk1, PCNA, and SP1 were measured by Western blotting. HSP70 served as a loading control.
(B) Scanning densitometry analysis of SP1, phospho-ATM, Chek1, SP1 and PCNA. Eto, etoposide-treated.

Figure S3

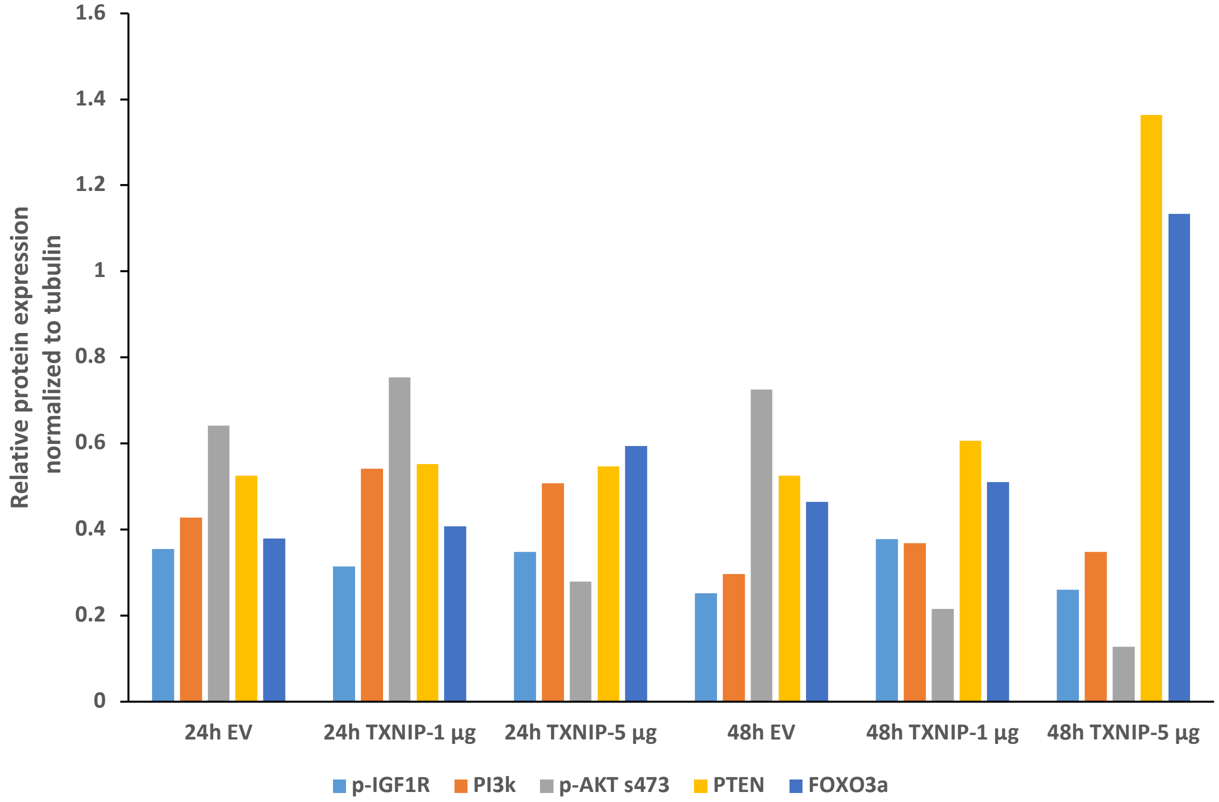

Figure S3. Scanning densitometry analysis of the effect of TXNIP expression on the IGF1 signaling pathway. TXNIP-KD HEK293t cells were transfected with 1 or 5 μg of a TXNIP-GFP expression vector (or empty vector, EV) for 24 or 48 h. Cells were then harvested and Western blots were performed as shown in Figure 3C. The expression levels of p-IGF1R, PI3K, p-AKT, PTEN and FOXO3a at 24 and 48 h are presented. The results represent the raw data.

Figure S4

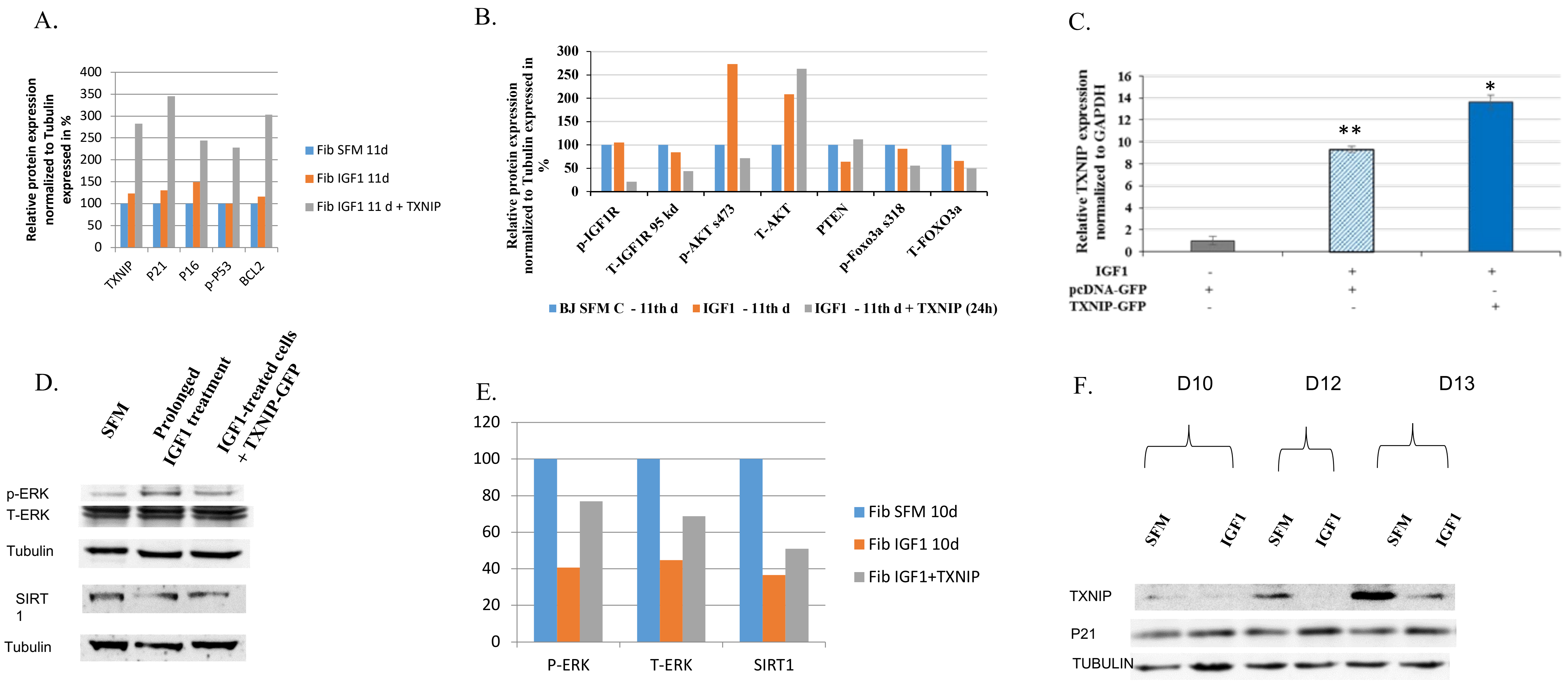

Figure S4. Effect of prolonged IGF1 treatment, or IGF1+TXNIP treatment, compared to untreated serum starved cells, on signaling mediators.
(A) Scanning densitometry of Western blot (Figure 4B) comparing expression of TXNIP, P53, BCL2, P21 and P16 relative to empty vector-transfected cells.
(B) Scanning densitometry of Western blot (Figure 4C) comparing expression of phospho- and total-IGF1R, AKT, FOXO3a, mTOR and PTEN.
(C) RT-QPCR analysis of *TXNIP* mRNA levels in IGF1- and IGF1+TXNIP-treated cells. *TXNIP* expression was normalized to *GAPDH* mRNA levels.
(D) Western blot analysis of phospho- and total-ERK and SIRT1 in IGF1- and IGF1+TXNIP-treated cells, compared to control cells. Tubulin served as control.
(E) Scanning densitometry analysis of Western blot shown in Figure S4D.
(F) Western blot of TXNIP and P21 in cells treated for 10, 12 or 13 days with IGF1.

Figure S5

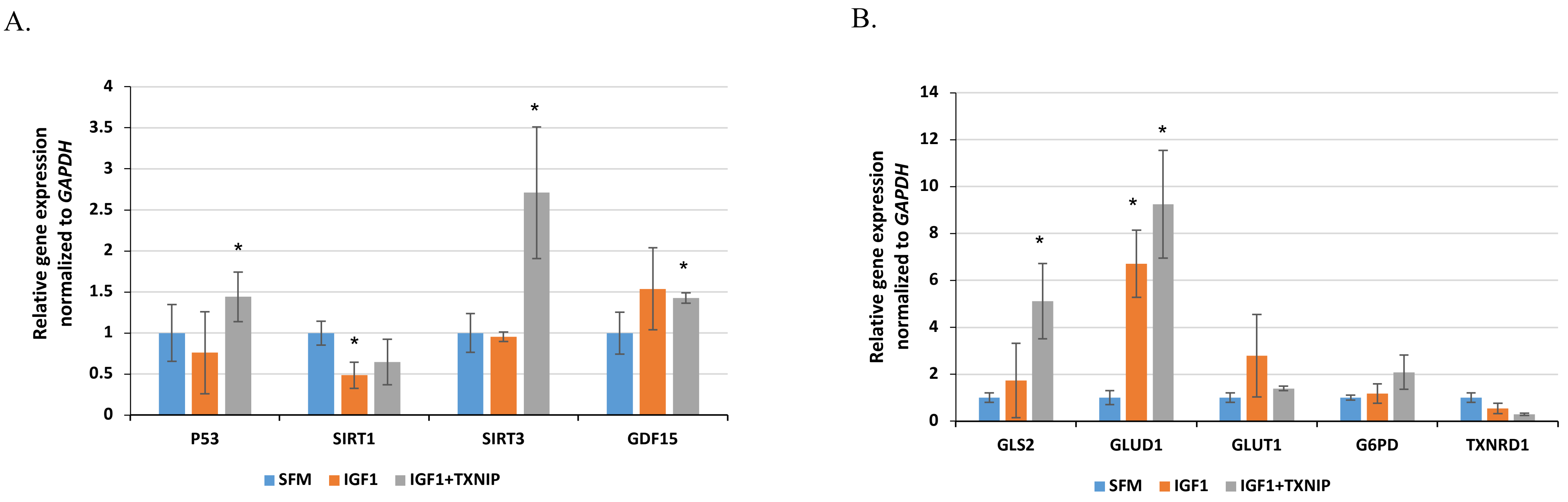

Figure S5. Effect of prolonged IGF1, or IGF1+TXNIP, treatment on selected gene expression

(A) RT-QPCR of *P53*, *SIRT1*, *SIRT3* and *GDF15* mRNAs.

(B) RT-QPCR of genes involved in glucose and glutamine metabolism (*GLS2*, *GLUD1*, *GLUT1*, *G6PD* and *TXNRD1*).

Gene expression was normalized to *GAPDH* mRNA levels. Bars represent the mean ± SEM of three independent experiments. A value of 1 was given to the mRNA levels in serum-starved cells transfected with empty vector.

Figure S6

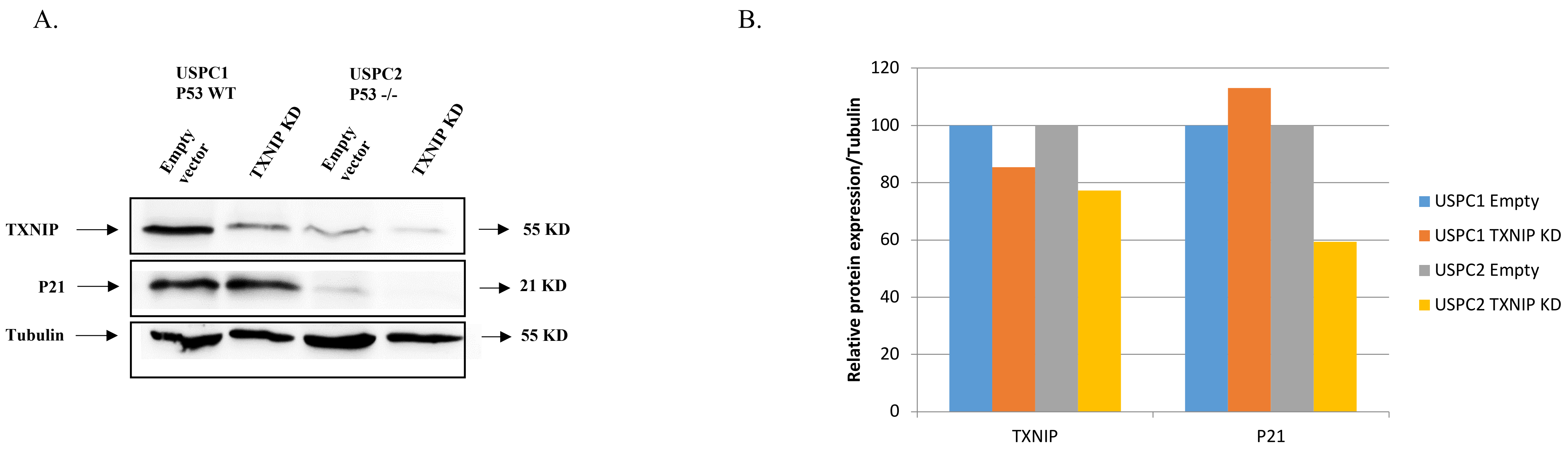

Figure S6. Effect of TXNIP KD in P53 mutated uterine cancer cells.

(A) Wild type P53-expressing (USPC1) and mutant P53-expressing (USPC2) uterine serous carcinoma cell lines were transiently transfected to TXNIP KD plasmid (or empty vector). Levels of TXNIP and P21 were measured by Western blots. Tubulin served as a loading control.

(B) Scanning densitometry analysis of TXNIP and P21 protein levels.

Figure S7

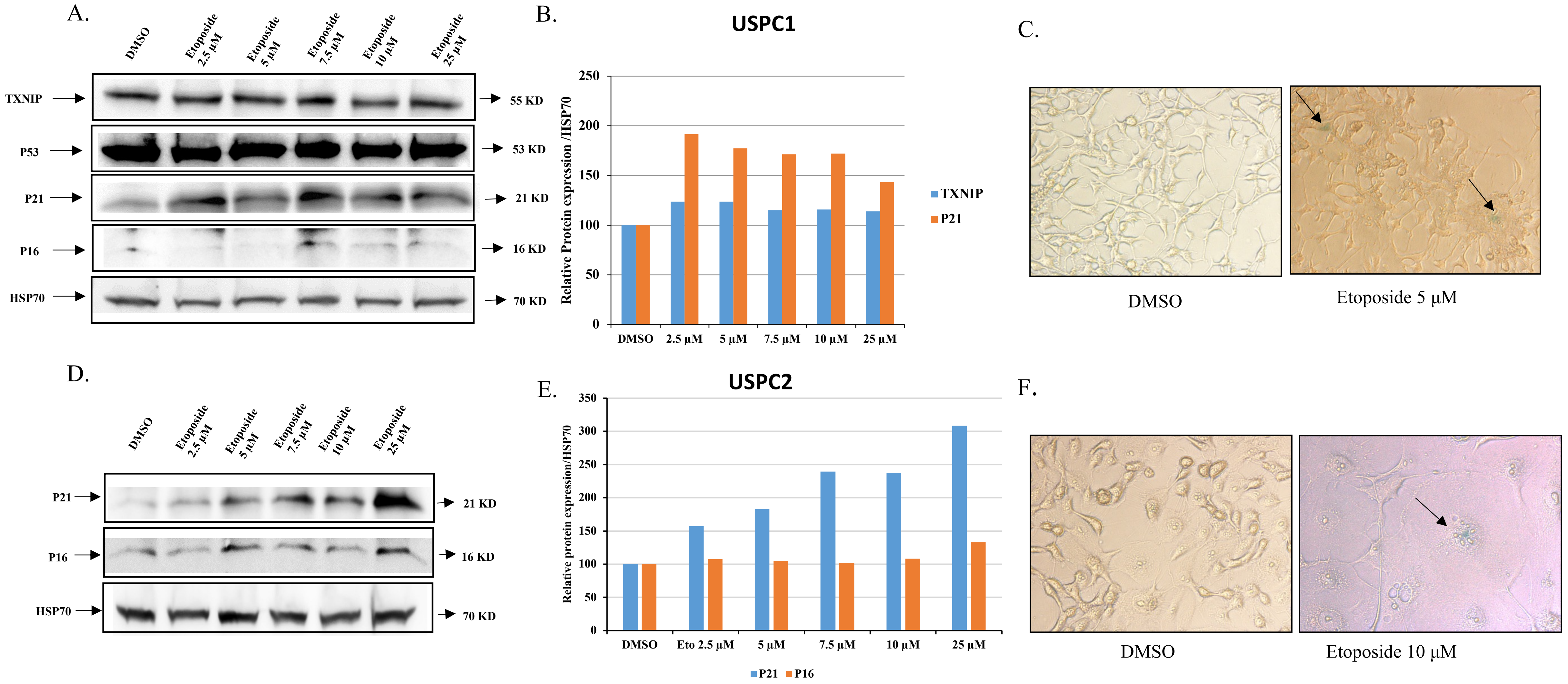

Figure S7. Effect of etoposide treatment on senescence induction in USPC1 (A-C) and USPC2 (D-F) uterine carcinoma cells.
(A, D) USPC1 (A) and USPC2 (D) cells were treated with increasing doses of etoposide (or DMSO, control) for 24 h, after which levels of TXNIP, P53, P21 and P16 were measured by Western blots. HSP70 served as a control.
(B, E) Scanning densitometry analysis of Western blots shown in panels A and D.
(C, F) Representative images of beta-gal staining: DMSO- (left) and etoposide- (right) treated cells were imaged through microscope after 48 h of senescence induction. Senescent cells are marked with black arrows.

Figure S8

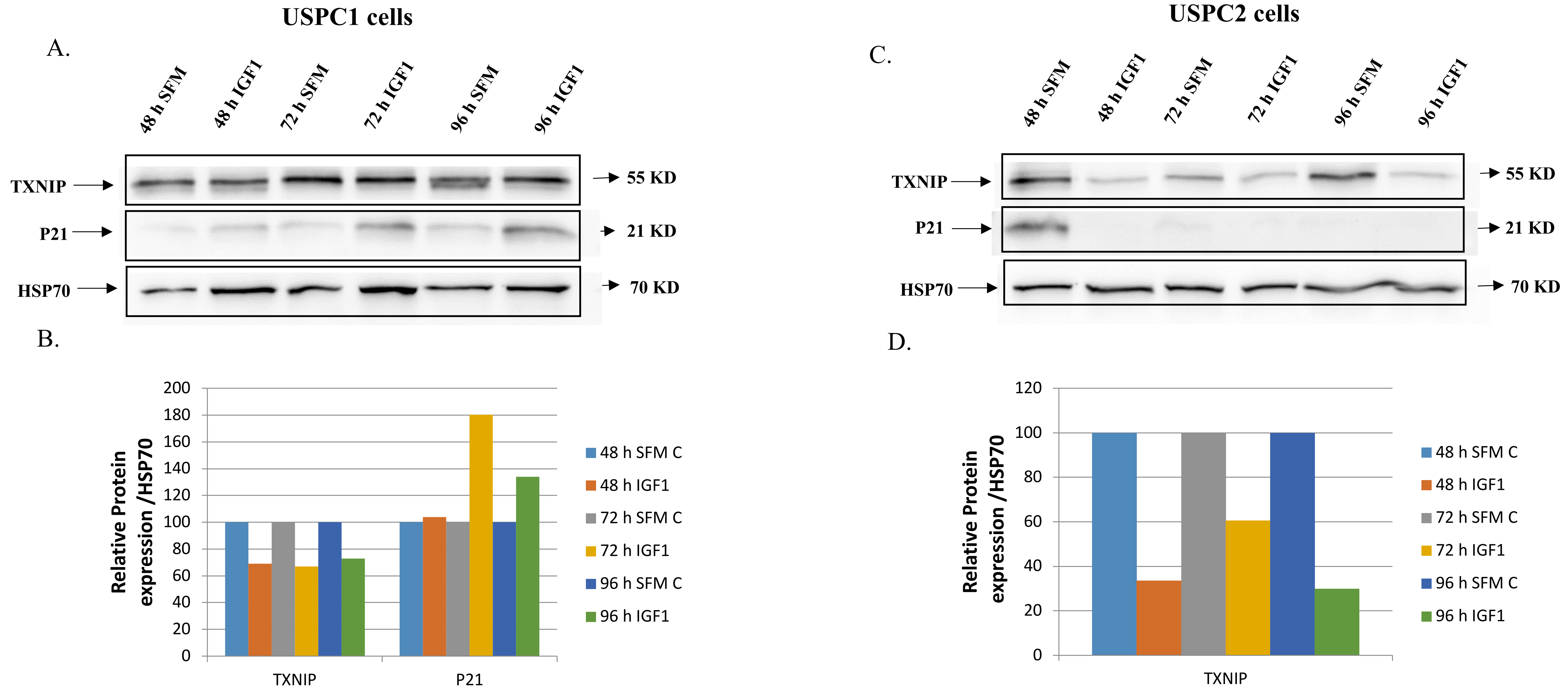

Figure S8. Effect of IGF1 on TXNIP expression in USPC1 and USPC2 cells.
(A, C) USPC1 (A) and USPC2 (C) cells were treated with IGF1 for 48, 72 or 96 h (or left untreated), after which TXNIP and P21 levels were measured by Western blots.
(B, D) Scanning densitometry analysis of Western blots shown in panels A and C.

Figure S9

A.

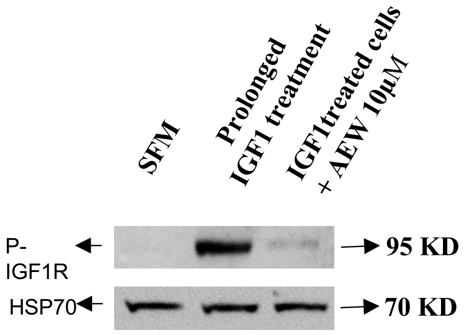

B.

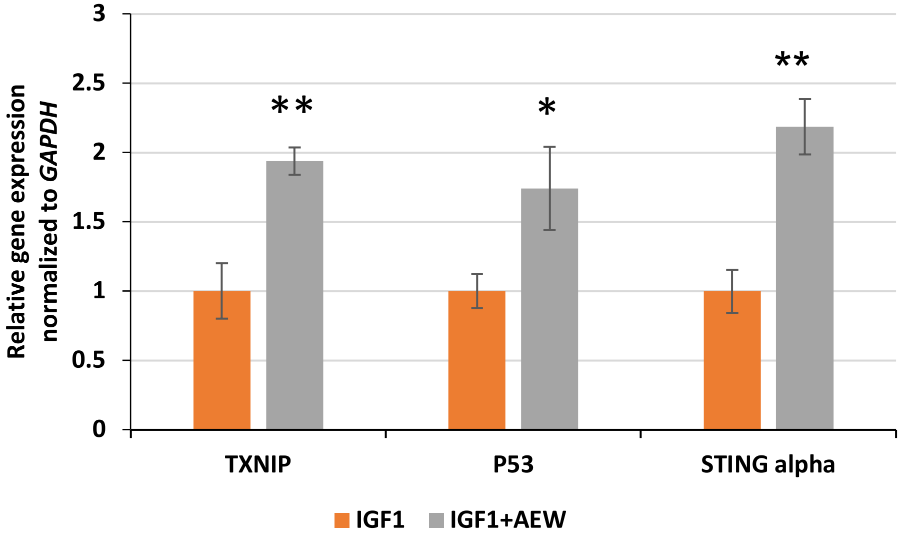

Figure S9. IGF1R activation sustains the inhibition of STING-alpha activation during prolonged IGF1-induced premature senescence.

(A) Fibroblasts were treated with IGF1 for 11 days in the absence (lane 2) or presence (lane 3) of an IGF1R inhibitor (AEW541) during the last 24 h. Serum starved cells (lane 1) were used as controls. Cells were then lysed and IGF1R activation was measured by Western blots.

(B) RT-QPCR of *TXNIP*, *P53* and *STING-alpha* mRNA levels comparing IGF1 treated and IGF1+AEW treated cells. Gene expression was normalized to *GAPDH* mRNA levels. Bars represent the mean ± SEM of three independent experiments. A value of 1 was given to the mRNA levels in IGF1-treated cells.

Figure S10

Long-term IGF1 stimulation leads to cellular senescence *via* interaction with the thioredoxin-interacting protein, TXNIP

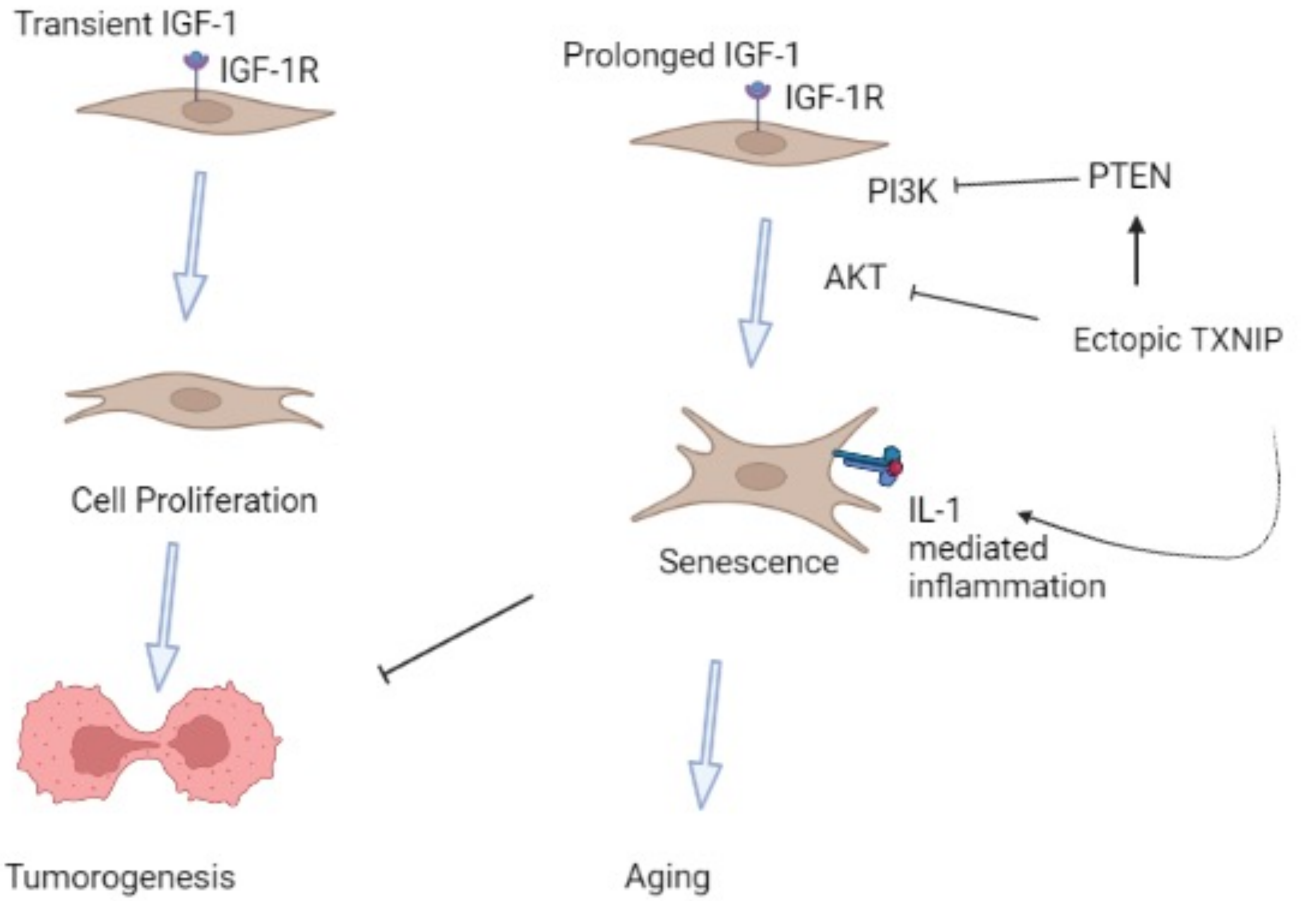

Figure S10. Schematic diagram of transient (short-term) versus prolonged IGF1 treatment.
Whereas short-term IGF1 stimulation is associated with cell proliferation and, potentially, tumorogenesis, long-term IGF1 stimulation leads to cellular senescence via interaction with TXNIP.
